# Advances in Wearable Technology: MXene-Based Multifunctional and Biomedical Smart Textiles

**DOI:** 10.1021/acsomega.5c08488

**Published:** 2025-12-29

**Authors:** Nishat Sarmin Rupanty, Joyjit Ghosh, Tasneem Noor, Tanvir Rahman Asif, Sayef Ahmed, Sadhin Howlader, Vladimir Reukov

**Affiliations:** † Department of Textiles, Merchandising, and Interiors, 1355 University of Georgia, Athens, Georgia 30602, United States; ‡ Department of Textile Engineering, 130048 Ahsanullah University of Science and Technology, Dhaka 1208, Bangladesh

## Abstract

The two-dimensional transition metal carbides, nitrides, and carbonatites known as MXenes have become a revolutionary class of materials for developing multipurpose wearable electronic fabrics, or e-textiles. Their remarkable mechanical flexibility, hydrophilicity, customizable surface terminations, and electrical conductivity make them perfect for incorporation into a variety of textile substrates. This review paper provides a thorough examination of MXene structures, synthesis pathways, and surface chemistry, emphasizing how these properties affect performance in textile applications. Scalability, homogeneity, and durability are evaluated for a variety of integration techniques, including dip coating, spray coating, printing, electrospinning, layer-by-layer assembly, and composite production. A wide range of applications, such as extremely sensitive strain and pressure sensors, energy storage and harvesting devices, electromagnetic interference (EMI) shielding, thermal management systems, antimicrobial and medical textiles, and communication interfaces, demonstrate the versatility of MXene-based e-textiles. In addition to durability issues, including oxidation resistance, wash stability, and mechanical robustness, special emphasis is placed on performance parameters such as conductivity, gauge factor, shielding effectiveness, and thermal response. Lastly, the paper outlines potential approaches to creating sustainable, biocompatible, and commercially viable MXene-integrated textiles, while discussing existing limitations, including cytotoxicity, environmental stability, and limitations of large-scale production. Through the integration of materials science, textile engineering, and application-driven design, MXenes has the potential to transform the next generation of innovative fabrics for consumer electronics, healthcare, and defense.

## Graphical abstract

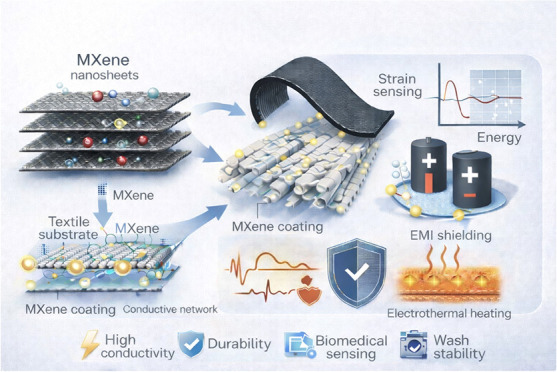

## 1 Introduction

Wearable electronic textiles, commonly known as e-textiles or bright fabrics, have rapidly gained popularity as a transformative technology because of the intersection of textiles and electronics. Their on-body systems, which offer real-time monitoring, communication, and interactive feedback, have increased the global demand.^1^ These fabrics integrate electronic functions such as sensing, motion monitoring, power generation, and data transmission directly into fibers or fabric structures, enabling seamless interaction between the computer and the human body while maintaining the look and feel of conventional clothing.^2^ As a result, demand for these is increasing in various sectors. The primary uses of wearable electronic textiles can be found in healthcare to monitor vital signs like heart respiration rate, pulse and monitor temperature as well as to physiological data (ECG, EEG, EMG etc.); they also play a key part in **defense by** enhancing soldier safety through temperature regulation, superior infrared camouflage and physiological surveillance and in **consumer electronics**, they support functions like activity tracking and movement control.^3–5^ E-textiles are not like traditional wearables such as wristbands or chest straps; they are designed to be flexible, stretchable, breathable, and lightweight to ensure long-term comfort for users even during continuous motion or extended wear.^6^ This integration also needs to fulfill fabric qualities such as drapability, softness, and washability, while one disadvantage remains as conductive or functional materials that do not degrade over time.^7,8^ From the concepts of metallic threads, smart textiles have evolved significantly into advanced nanomaterial-based systems. In the initial phase, fabric was integrated with metallic wires and coated yarns to provide conductivity. Though these materials were functional, they had significant drawbacks, including rigidity, heaviness, limited stretchability, and discomfort for end-users, especially during movement.^9^ Moreover, their incompatibility with textile flexibility hindered large-scale adoption in wearable applications. In the meantime, people’s interest in e-textiles is rising quickly, and the demand for lightweight, stretchable, and multifunctional materials is increasing exponentially. Significant advancements can be seen when conductive polymers and nanomaterials, particularly carbon-based structures such as carbon nanotubes (CNTs), graphene, and silver nanowires (AgNWs), are integrated. These materials significantly improve weight, durability, flexibility, and surface-to-volume ratios; at the same time, they become essential for applications such as biosignal detection and energy harvesting.^5,10^ However, some challenges related to biocompatibility and environmental stability remain a question mark on this technological advancement.^11^

Although the application of nanomaterials has enhanced the functionality of smart textiles, it cannot be used widely due to fundamental performance and safety limitations. To overcome these drawbacks, many conductive fillers, such as carbon nanotubes, graphene, and silver nanowires, have been developed that exhibit high electrical conductivity but suffer from performance degradation and microcracks under repeated flexing or stretching.^12^ Their interfacial adhesion with textile fibers was weak, which exacerbates poor bonding results in delamination during laundering or wear.^13^ Techniques such as electrospinning, Chemical Vapor Deposition (CVD), atomic layered deposition, etc., have been invented to resolve concerns around scalability, but still pose a significant barrier. Contrarily, they are effective at the lab scale but incompatible with industrial roll-to-roll textile processing, often raising production costs and equally pressing toxicity concerns. Some nanomaterials, such as metal oxides, fullerenes, graphene, and surface-modified CNTs, have demonstrated cytotoxicity or environmental effects, posing significant concerns for both human health and ecological impact.^10,14–16^ New products have been introduced daily, focusing on materials that simultaneously meet multiple demands, such as stretchability, breathability, durability, and functionality. One promising candidate is MXenes, which are two-dimensional transition metal carbides, nitrides, and carbonitrides obtained by removing the A-layer from MAX phases via various etching methods.^17^ These materials exhibit unique properties, including high electronic conductivity, hydrophilicity, excellent flexibility, and ion intercalation, which are also compatible with scalable fabrication techniques, and they can form a strong bond with soft textile substrates.^18,19^ They have an exceptional layered architecture, and due to this structural configuration, electric current can easily pass through MXenes, which is often comparable to many traditional carbon-based nanomaterials such as graphene oxide.^20,21^ They have notable advantages for wearable electronics, as they are flexible and hydrophilic. These features allow them to mix well in water and readily coat different types of fabrics, which is not often found in other 2D materials^22–24^ Because of these characteristics, MXenes can be applied with simple methods like dip-coating, inkjet printing, or screen printing, making them great for creating soft, durable, and washable interfaces.^25^ They also have a high surface area that can be modified for various applications, from sensors to energy storage, making it breathable, EMI shielding, and their intrinsic compatibility with both natural and synthetic fibers shows remarkable potential for the next generation of electronic fabrics.^26,27^

This review aims to explore the contribution of MXenes in both fundamental and applied domains and critically examine the evolving role in the development of wearable electronic textiles (e-textiles). Starting with analyzing the structural, synthesis techniques, electronic, mechanical, and surface chemical compositions that distinguish MXenes from other nanomaterials. The study also addresses dip-coating, spray deposition, printing, and fiber spinning, like diverse integration techniques, and highlights how these processes influence the properties of MXenes. To draw attention to MXene’s multifunctional potential, applications are categorized across biosensing, energy storage, electromagnetic interference (EMI) shielding, electrothermal actuation, and medical diagnostics.

## 2 MXenes: Structure, Synthesis, and Properties

### 2.1 Structural Characteristics

MXenes are a unique class of two-dimensional (2D) transition metal carbides and nitrides derived from ternary MAX phases, which can be represented by the formula M_*n*+1_X*_n_* T_*x*_ where M refers to an early transition metal, X stands for carbon and/or nitrogen, T_*x*_ denotes surface terminations (−OH, −F, =O) and *n* is the number of layers of X between two layers of M.^28,29^ In aqueous media, these terminations play a central role in determining the electrochemical behavior and surface energy of MXenes.^30^ Akin graphite, MXenes exhibit lamellar morphology consisting of atomic layer structure held by van der Waals forces, where (*n*+1) layers of “M” atoms alternate with “*n*” layers of “X” atoms. This layered structure enables MXenes to intercalate and transport ions smoothly, which is essential for energy and sensing applications.^31^ Functional groups like −O, −OH, −F, etc., terminate the surface of MXenes, and these terminations are byproducts of the chemical etching process used to produce MXenes. The presence and type of these terminations influence the chemical reactivity and properties of the MXenes, such as hydrophobicity and electrical conductivity.^32,33^

### 2.2 Common and Emerging Compositions

Among the various MXenes, for example, Ti_3_CNT_*x*_, Ti_2_CT_*x*_, Ti_4_N_3_T_*x*_, etc., synthesized to date, Ti_3_C_2_T_*x*_ is indeed the most studied composition, well-known for its excellent ion movement and mechanical properties.^34^ Through selective etching of the aluminum layer, **Ti**_**3**_ **C**_**2**_ **T**_**x**_ is typically derived from the MAX phase of Ti_3_AlC_2_ and forms a 2D structure layer with terminal groups (−OH, −O, −F).^35^ A graphene/PDMS-based strain sensor, Ti_3_C_2_T_x_, has been developed to detect electrical ion currents and capture various physiological signals.^36^ However, attention to other MXene compositions, such as Mo_2_TiC_2_, Nb_2_C, and V_2_C, is growing rapidly due to their emerging **multifunctional properties**. For example, the resistance of these Mo-based MXenes increases with decreasing temperature, exhibiting semiconductor-like behavior.^37^ While **Ti**_**3**_ **C**_**2**_ **T**_**x**_ is a single-transition-metal MXene, Mo_2_TiC_2_ is a double-transition-metal MXene, which demonstrates higher volumetric capacitance, making it valuable in both energy storage and optical applications.^38^ Similarly, **Nb**_**2**_ **C** can promote wound healing without inducing fibrosis, and its micro- and nanostructures promote cell adhesion and proliferation, while simultaneously possessing photothermal properties.^39,40^ On the other hand, **V**_**2**_ **C** is flexible and accelerates chemical reactions without permanently changing itself. Additionally, V-based MXenes, such as V_1.8_Nb_0.2_CT_*x*_, have ultrahigh volumetric capacitance of 1698 F/cm^3^ at a scan rate of 2 mV/s.^41^ These two compositions (**Nb**_**2**_ **C** and **V**_**2**_ **C**) have opened new pathways for integrated wearable sensors and self-healing textiles.

### 2.3 Synthesis Techniques

Generally, top-down approaches are used to synthesize MXenes, in which the A-layer element is selectively etched from a MAX phase using hydrofluoric acid (HF) or in situ-generated etchants, such as LiF–HCl.^35,42^ HF-Based Chemical Etching, first introduced by Naguib et al. (2011), concentrated hydrofluoric acid is used to etch out Aluminum (Al) from Ti_3_AlC_2_ MAX phase:

$${\mathrm{Ti}}_{3}{\mathrm{AlC}}_{2}+3\mathrm{HF}\to {\mathrm{AlF}}_{3}+{\mathrm{Ti}}_{3}C_{2}+1.5H_{2}↑$$

Following etching, Ti_3_C_2_ is terminated by surface groups (−O, −F, −OH) through reactions with water and HF.

$${\mathrm{Ti}}_{3}C_{2}+2H_{2}O\to {\mathrm{Ti}}_{3}C_{2}{\left(\mathrm{OH}\right)}_{2}+H_{2}↑$$

$${\mathrm{Ti}}_{3}C_{2}+2\mathrm{HF}\to {\mathrm{Ti}}_{3}C_{2}F_{2}+H_{2}↑$$

The resulting MXenes are multilayered structures with −OH, −F, and −O functional groups. Among these, LiF–HCl can be adjusted under milder conditions than the others and forms larger, more stable flakes suitable for coatings.^43,44^ However, in these traditional methods, hazardous and toxic fluoride-containing reagents are often used. As a result, they limit large production volumes.^45^ To address these challenges, safer and more scalable synthesis techniques have been developed. Although bottom-up strategies such as chemical vapor deposition (CVD) and molten salt synthesis are less explored than top-down methods, they offer promising alternatives with precise control over surface termination. The Lewis acidic molten salt method has an advantage as it can be applied to numerous MAX materials. Due to its universal and easy etching procedure, large-scale production is possible. It also provides better control of surface terminal groups and ensures metal nanoparticles can deposit uniformly.^46–48^ Unfortunately, both of these are very complex procedures, and their high cost is a significant concern. Another innovative technique for producing MXenes free of fluorine is the mechanochemical route.^45^ With etchant NH_4_HF_2_, a new ultrafast Low-Temperature Molten Salt (LTMS) has recently been developed that can produce various MXenes within minutes and generate more than 100g of Ti_3_C_2_T_*x*_ in a single reaction.^49^ Furthermore, a molten salt-shielded synthesis (MS^3^) method is capable of rapid synthesis of MXenes in open air, using Lewis-acid salts as etchants^34^

### 2.4 Surface Chemistry and Tunability

MXenes can be modified and controlled to achieve desired surface chemical properties and functionalities. This tunable surface chemistry is enabled by their terminal groups (−OH, −O, −F), which also significantly influence interactions with other materials, such as polymers or textiles. One significant advantage of these surface terminations is that they can be controlled during or after synthesis with slight modifications, allowing tailored functionalization.^50–53^ However, MXenes made using traditional methods face challenges in enabling postsynthetic modifications because of strong chemical bonding between surface metal atoms and oxygen or fluorine.^52^ It is established that the surface chemistry of MXenes plays a paramount role in their properties and interactions, where O-terminated MXenes generally show higher reactivity and adsorption capacity compared to other terminated groups (F– or Cl−).^54^ By controlling these surface terminations, the performance of MXene in applications such as energy storage, gas sensing, and catalysis can be enhanced.^55–57^

### 2.5 Key Properties for Textiles

Among various materials, MXene-based fibers and textiles deliver some exceptional properties. For this reason, they have become highly suitable candidate for advanced textile applications. One key feature is that electrical current can flow easily through MXene-coated cellulose hybrid fibers and MXene fibers, with conductivities of 0.06 and 3637.9 S/cm, respectively.^58,59^ This high conductivity opens new opportunities in flexible electronics and electromagnetic interference protection.^60,61^ Besides, they can deform and reform under stress, rather than completely dissolve in water. Owing to their mechanical flexibility and aqueous dispersibility, MXenes can be applied via wet-spinning59 and dip-coating62, among other fabrication methods. These processes also increase the fiber’s tensile strength (150.7 MPa) without making it rigid, making it suitable for weaving and knitting.^59^ However, there is one drawback: MXene can compromise electrical conductivity. Interestingly, by combining with polymer-like short-chained hemicellulose, mechanical properties can be enhanced without significantly compromising it.^62,63^ MXene-based textiles are also suitable for heat-related applications due to their excellent photothermal behavior. For example, Silk is the world’s most favored textile material because of its luster.^64^ This Silk fabric coated with MXene exhibits satisfactory photothermal and electrothermal properties by converting light and electrical energy into heat. It rapidly responds to the energy and can function for a long time.^65^ Additionally, MXene aerogel fibers can absorb a remarkable amount of light and have high electrical conductivity. Consequently, they can respond to both light and electrical stimuli quickly.^66^

Table 1 presents a comprehensive and well-structured summary of MXene compositions, synthesis methods, and distinctive features and properties. This overview facilitates a clear understanding of the various types of MXenes and their potential applications.

**Table 1:** Summary of MXene Compositions, Synthesis Methods, Features and Properties

Synthesis Methods	MXene Composition	Mechanism	Key Features	Properties	refs
HF Etching (Top-Down)	-Ti_3_C_2_T_*x*_	-Selective removal of Al layer using HF acid	-High conductivity	-Electrical conductivity (∼10,000 S/cm)	Lim et al.^67^
			-Flexible		
			-Scalable production	-EMI shielding	
Molten Salt Etching (ZnCl_2_ Method)	-Ti_3_C_2_T_*x*_	-Selective A-layer removal in molten ZnCl_2_ salt at 600 °C	-Chlorine-terminated MXene	-Stable in air	Kruger et al.^46^
	-Mo_2_TiC_2_T_*x*_		-Improved stability	-Potential for sensors and batteries	
LiF-HCl Etching (Mild Top-Down)	-Ti_3_C_2_T_*x*_	-In-situ HF generation via LiF + HCl, selective Al etching	-Mild synthesis	-High dispersibility	Gogotsi & Huang^68^
			-Fewer defects	-Mechanical strength	
	-Nb_2_CT_*x*_		-Good flake quality		
Fluorine-Free Mechanochemical Etching	-Ti_3_C_2_T_*x*_	-Mechanical ball milling with Lewis acidic salts, removing the layer	-Fluoride-free	-Sustainable synthesis	Rokovic et al.^45^
	-V_2_CT_*x*_		-Environment-friendly	-Promising for bioapplications	

### 2.6 Comparison with Other Nanomaterials

Compared with nanomaterials such as graphene, carbon nanotubes (CNTs), and silver nanowires, MXenes exhibit strong hydrophobicity, flexibility, and mechanical strength, as well as unique electrical, magnetic, and catalytic properties.^18,32,61,68^ They can also form diverse nanocomposites with other materials, thereby increasing their versatility.^69^ Because of their hydrophobic nature, MXenes have better dispersibility than graphene and CNTs in aqueous solutions. In contrast, graphene tends to agglomerate within the polymer, limiting its ability to strengthen the fiber.^24,70^ MXenes also offer safer handling than CNTs and exhibit effectiveness against a wide range of bacteria. A useful compromise between graphene oxide (GO) and carbon nanotubes (CNTs) is provided by reduced graphene oxide (RGO). Similar to GO, RGO is produced via graphite oxidation; however, partial reduction recovers a significant portion of the sp2 network, resulting in superior electrical conductivity, stiffness, and a smaller interlayer gap. While CNTs remain superior in intrinsic tensile strength and one-dimensional transport, RGO provides a two-dimensional, high-surface-area platform that is easy to produce into films or composites. Scalable and economical production is advantageous to RGO; the reduction process determines the quality. RGO is a flexible addition to GO and CNTs because applications frequently require a balance among conductivity, mechanical reinforcement, and solution processability (e.g., transparent conductors, energy storage, and composites).^71–74^ This multifunctionality of MXenes gives them an edge compared to other nanoparticles.^69,75,76^

## 3 Integration Strategies in Textiles

### 3.1 Fabric Surface Modification

The rapid emergence of MXenes is gaining worldwide attention, as is the need for their implementation in textiles. Techniques such as surface treatments, coatings, printing, and functionalization can enhance bonding between MXene and the fabric, making the composite more robust and long-lasting. By modifying the fabric surface, the attraction between fibers and MXenes significantly improved.^77^ For instance, physical methods such as corona discharge and plasma treatment have already proven effective in altering surface structure and enhancing the hydrophilicity and wettability of fabrics.^78,79^ Furthermore, Vapor-phase atomic layer deposition, surface grafting, sol–gel, etc., are established chemical methods for surface modification to improve MXene integration. Interestingly, surface modification offers various advantages but also has a few drawbacks that directly affect the properties of the resulting textiles. For example, treatment with citric acid, Quat 188, and GPTMS has adversely affected the antibacterial activity and UV protection of the nanocomposite-modified cotton fabric.^80–82^ Hence, a combination of physical and chemical surface modification techniques can be employed to optimize the integration of MXenes onto textiles. This selection of appropriate methods depends on the desired properties of the final product.

### 3.2 Coating and Deposition Methods

The challenges of applying MXenes to fabric surfaces remain significant; one key issue is achieving durable adhesion between MXene flakes and the inherently flexible, chemically inert fabric fibers. The weak interfacial bonding often results in poor wash fastness and limited long-term stability under mechanical deformation.^83–86^ Additionally, MXenes are prone to oxidation in air or aqueous environments, leading to degradation of their structural integrity and loss of functionality over time. The uneven surface morphology of textile substrates further complicates uniform MXene coating, causing nonhomogeneous layer deposition and inconsistent performance across the fabric.^87^ Moreover, maintaining the intrinsic conductivity of MXenes during large-scale coating or finishing processes—such as dip-coating, padding, or spray deposition—remains difficult due to process-induced aggregation or chemical changes.^88,89^ These challenges collectively hinder the scalability and practical integration of MXene-based functional textiles for wearable and smart material applications.^74^ To solve this remotely, several deposition methods such as Dip-coating, Spray-coating, Vacuum filtration, etc. have been explored. Surprisingly, dip-coating is noted as an effective and scalable technique for coating textile substrates. In dip-coating, a substrate is immersed in a solution to create a uniform composite layer and ensure good adhesion (Figure 1).^90^ Here, with the help of a recently developed automated yarn dip coater, high-quality MXene-coated yarn can be produced on a large scale. This innovative approach also improved yarn performance, with lower electrical resistance, superior uniformity, and reduced material consumption compared to traditional manual techniques.^91^ Another excellent method that offers precise control over MXene deposition, with the ability to follow patterns, is Spray coating. Initially, a homogeneous mixture of MXene dispersion is prepared and applied to electrically conductive polyaniline/cotton fabrics using a spray nozzle. After spraying the solution onto the textile substrate, the fabric was left to dry, allowing the MXene nanosheets to adhere to the fibers.^21,92,93^ Fabric coating structure can also be optimized using this technique. Additionally, the vacuum-assisted filtration method can effectively produce uniform MXene thin films at the lab scale.^94^ Though it is not explicitly with textiles, this technique could be a potential way to coat the fabric with highly controlled MXene layers. Interestingly, new integration techniques have been developed by combining multiple coating methods, such as dip-dry coating and electroless plating. Figure 1 depicts the process of developing MXene/Ni-coated polyester fabrics, where the PET fabric was first fabricated using a dip-dry coating process, and MXene nanosheets were applied to the fabric. Further, a layer of nickel was added through electroless plating, resulting in a double-layered structure. Increasing the number of dip-dry coating iterations leads to superior electrical conductivity (up to 113.8 S/cm), hydrophobicity, and electromagnetic interference (EMI) shielding effectiveness, while maintaining constant porosity, compared to either MXene or Ni-coated polyester fabrics.^93,95^

**Figure 1:**
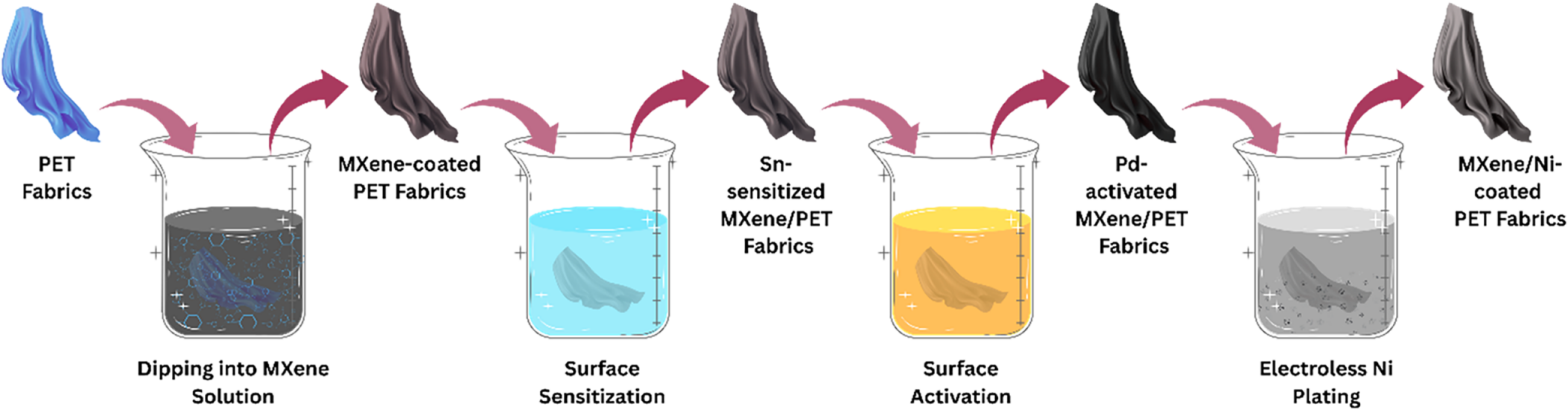
MXene/Ni coating process on PET fabrics (created with MS PowerPoint).

### 3.3 Printing and Patterning

MXene’s exceptional electrical conductivity, dispersion quality, hydrophilicity, and rheological properties have made it a potential ink material for printing applications. Inkjet and screen printing techniques have successfully fabricated MXene-based circuits and sensors. In the lab, inkjet printers are widely used for research and operate on the drop-on-demand (DOD) principle, using piezoelectric or thermal mechanisms to generate pressure via an electric field and eject ink droplets from a nozzle onto a substrate.^96,97^ In a study on Tunable capacitance in all-inkjet-printed nanosheet heterostructures, ten Elshof et al.^98^ illustrate all-inkjet-printing wherein water-based MXene ink without an additive was inkjet printed on thin films as electrodes, afterward hydrated graphene oxide (GO) nanosheets, a water-based electrolyte (ink) was inkjet over the top of it to form an all-solid-state MSC. Again, MXene electrodes were inkjet-printed on top of MSC to complete the fabrication of an all-solid-state SSC, as shown in Figure 2a. Inkjet-printed MXene films and MXene ink have demonstrated remarkable results in creating skin-conformable electronics and multifunctional biosensing units. These printed MXene films can detect electrocardiographic signals and sweat ions (Na^+^) while exhibiting excellent flexibility and long-term stability.^99^ Specially developed MXene/xanthan gum hybrid ink for screen printing has also shown promising results in producing high-conductive films (up to 4.8 × 10^4^ S/m), suitable for piezoresistive sensors, Joule heaters, and electromagnetic shielding.^96^ In screen printing, the rheological properties of inks are more essential than in injection, as high-viscosity inks are squeezed through a patterned stencil screen onto substrates. The two primary methods are flat-bed, in which ink is pressed through a flat screen, allowing multilayer deposition, and the rotary approach, which uses a polyester screen cylinder or perforated metal (Figure 2).

**Figure 2:**
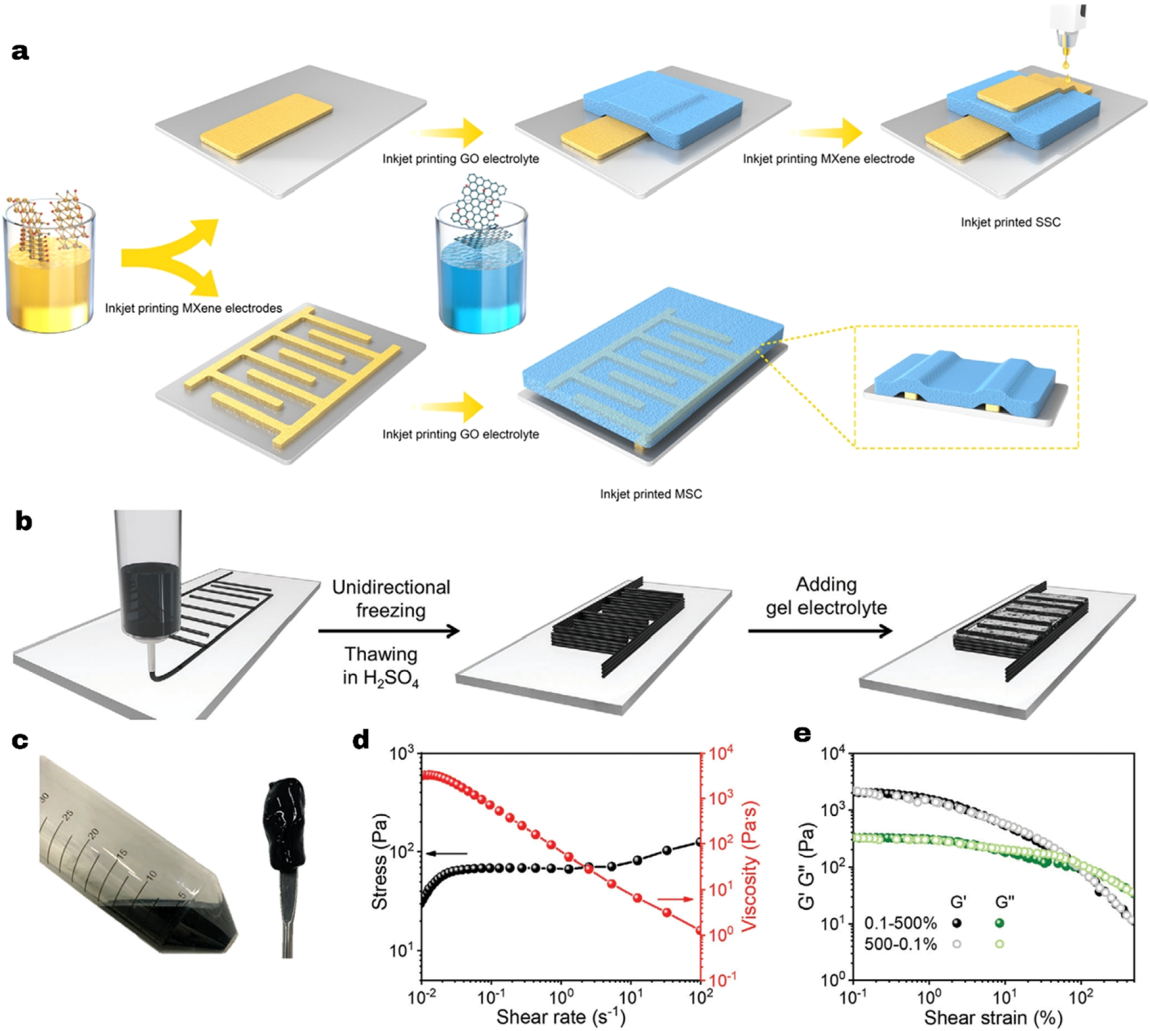
(a) Schematic illustration of all-inkjet-printing-based heterostructure symmetric supercapacitors (SSC) and microsupercapacitors (MSC). Reprinted with permission from ref 98. Copyright [2021] [Elsevier]. (b) The fabrication process of 3D-printing all-MXene MSC via MSES. (c) The digital photographs of the MXene slurry. (d) Shear-thinning behavior of the MXene inks (e) The oscillatory measurements of the MXene ink. Reprinted with permission from ref 100. Copyright [2021] [Wiley].

While inkjet and screen printing are well-established techniques for integrating MXenes, direct writing and 3D printing are emerging approaches for creating 3D, custom-shaped MXene architectures with functional gradients. Huang et al.^100^ Proposed a promising approach that leverages the weak gelation properties of MXene slurry to form oriented microstructures, even at high MXene concentrations. This 3D printing strategy can also be applied to the additive manufacturing of MXene microsupercapacitors (MSCs), as demonstrated in Figure 2b, where the MXene slurry forms a viscous ink without aggregates Figure 2c,d shows the shear-thinning thixotropic behavior of inks (stress and viscosity against different shear rates) that reflects that, when the shear rate increases, the viscosity of MXene ink decreases.^101^ Additionally, Figure 2e presents the oscillatory measurements (1 Hz) of the MXene ink, which sweeps from 0.1 to 500% and back to 0.1% strain. The ink for this integrated system is water-based and additive-free, capable of high-resolution printing at room temperature. Besides, these advanced printing methods offer wireless communication and intelligent sensing, enabling 3D microelectronic circuits, which are essential for energy storage applications.^102,103^

### 3.4 Composite and Hybrid Systems

The successful deployment of MXenes in wearable electronics crucially depends on their integration into composite and hybrid structures. Composite strategies, such as MXene–polymer blends, are a promising approach, with polymers such as polyurethane (PU) and Poly­(vinyl alcohol) (PVA) serving as flexible matrices. MXene-PU composites are commonly processed via dip-coating or electrospinning, and, interestingly, the resulting material exhibits excellent strain detection while retaining elasticity. In electrospinning, a solution of MXene nanosheets is dispersed in a PU solution, and then, under high voltage, the liquid is solidified into nanofibers. Azoan In contrast, MXene–PVA composites are prepared via solution casting, where Ti_3_C_2_T_*x*_ and PVA functional groups interact with the fabric’s polar groups via hydrogen bonding, enabling stable coatings on both woven and nonwoven substrates.^104,105^ Beyond single-matrix systems, MXene is combined with other nanostructures, such as graphene, carbon nanotubes (CNTs), or biopolymers, to create multifunctional materials. Using a layer-by-layer assembly or vacuum filtration process, a hybrid composite, MXene-graphene, was deposited with high efficiency for Joule heating.^106^ In another work, waterborne polyurethane (WPU) composites containing Ti_3_C_2_T_*x*_ MXene and functionalized carbon nanotubes (CNTs) exhibited exceptional anticorrosion performance when coated on copper substrates, with the optimal composition of 0.95 wt % Ti_3_C_2_T_*x*_ MXene and 0.05 wt % CNTs achieving the lowest corrosion rate of 2.1 × 10^–3^ μm/year. This opens a massive opportunity for smart textiles.^107^

### 3.5 Layer-by-Layer and Multi-Functional Architectures

The Layer-by-Layer method was introduced in 1992 as a cheaper and greener approach in which oppositely charged polyelectrolyte solutions or suspensions form a layer of cations and anions.^108^ The vacuum-assisted layer-by-layer assembly technique involves stacking MXene nanosheets with other materials, such as silver nanowires (AgNWs) or carbon nanotubes (CNTs), enabling conformal deposition of conductive substances onto textiles, creating a leaf-like nanostructure with enhanced properties.^109^ Here, a stacked layer is formed by placing multiple thin sheets of MXene on top of each other to enable synergistic effects. This method develops textiles with superhydrophobicity and humidity-sensing capabilities.^101^ For instance, integrating MXene/ZIF-8 via layer-by-layer assembly into cellulosic textiles yields outstanding antibacterial properties, as well as photothermal/photodynamic therapy activity.^110^ Similarly, the combination of MXene with polyaniline (PANI) prepared by a vacuum-filtration-assisted spray-coating method renders the textile responsive to acids and bases.^92^ These multifunctional architectures demonstrate the potential of a single textile platform for flexible sensors, energy-related properties, and EMI shielding.^13,111,112^

### 3.6 Post-Treatment and Wash Durability

Post-treatment can play a vital role in addressing durability and wash-resistance challenges in MXene-coated textiles. Using encapsulation techniques, MXene layers are coated with breathable polymers such as polydimethylsiloxane (PDMS) or polyurethane to provide an additional protective layer that safeguards against oxidation and mechanical degradation. Another practical approach is chemical cross-linking, in which a robust 3D network is formed when MXene’s surface reactive groups (−OH, −O, −F) bond with poly­(vinyl alcohol) (PVA), polyacrylamide, or graphene oxide, thereby enhancing interfacial adhesion and water/solvent resistance.^113,114^ Additionally, in another research, incorporating functional binders (e.g., using Polylactic Acid (PLA)) and a conductive filler (e.g., graphene nanoplatelets) in ink emulsion raises the ink viscosity, reduces woven structural mobility, makes the fabric stiffer, and remarkably increases the moduli of deformation of cotton fabric without sacrificing conductivity.^115^ When optimized, these strategies minimize delamination and preserve electrical performance after repeated washing and bending cycles (Table 2).

**Table 2:** Properties of MXene-Based Compositions Applied with Different Methods

Composition^a^	Method	Structure design	Thickness [μm]	Conductivity [S/cm]	EMI SE [dB]	refs
Heat-treated Ti_3_CNT_*x*_	Vacuum filtration	Porous	40	1786	116.2	Iqbal et al.^116^
Ti_3_C_2_T_*x*_/Cellulose	Vacuum filtration	Layered	12.1	1672	59.8	Freng et al.^117^
Ti_3_C_2_T_*x*_/Ni	Dip-dry coating	Double-layered	30	∼113.8	∼35.7	Jeong et al.^95^
Ti_3_C_2_T_*x*_/PET substrate	Inkjet printing	layered	1.35	1080	50	Demirel et al.^118^
Ti_3_C_2_T_*x*_/Xantham gum	Screen printing	Layered	12	480	40.1	Wen Li et al.^96^
Ti_3_C_2_T_*x*_/CNF	Electrospinning	Sandwich	150	29.8	55.4	Oliveira et al.^119^
Ti_3_C_2_T_*x*_/AgNW@silk fabric	Self-assembly	Layer by Layer	480	0.8 Ω sq–1	∼90	Zhen Yu et al.^109^
Ti_3_C_2_T_*x*_/PEI-APP@cotton fabric	Self-assembly	Layer by Layer	6.7	31.0	-	Xing et al.^120^
Ti_3_C_2_T_*x*_/PVA	Solution casting	Multilayered	27	7.16	44.4	Wang et al.^121^
Ti_3_C_2_T_*x*_/CNT	Freezing	lamellar and porous	3000	9.43	103.9	Koo et al.^122^

a Ni—Nickel; PET—Polyethylene Terephthalate; CNF—Cellulose Nanofiber; AgNW—Silver Nanowires; PEI—Polyacetimidate; PVA—Poly­(vinyl alcohol); CNT—Carbon Nanotubes.

## 4 Application Areas of MXene-Based E-Textiles

MXene-based e-textiles demonstrate remarkable potential across multiple domains due to their superior conductivity, flexibility, and tunable properties.^123^ Key applications include wearable energy storage (supercapacitors, batteries, and triboelectric nanogenerators), health monitoring (strain/pressure sensors, biosensors), and electromagnetic interference shielding for military/aerospace uses.^124,125^ They also enable thermal management (Joule heating/cooling), human-machine interfaces (gesture recognition, smart controls), and antibacterial medical textiles.^126,127^ Although smart wearables have significant potential, issues with scalability, durability, and environmental impact require further investigation to ensure commercial viability.^123^ MXene e-textiles’ versatility makes them a key component of future wearable technology (Figure 3).

**Figure 3:**
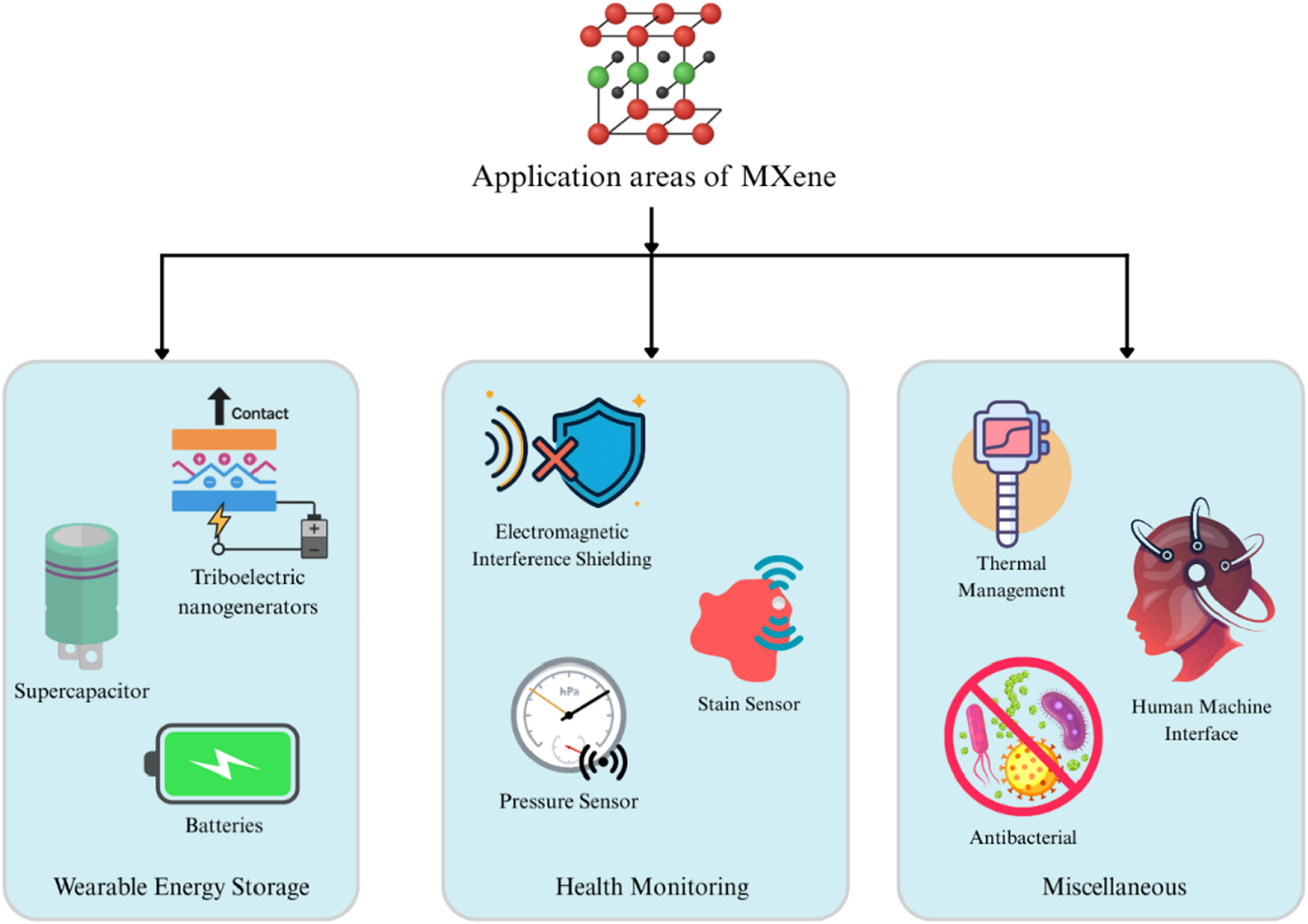
Diverse Applications of MXene: Innovations in Wearable Energy Storage, Health Monitoring, and Advanced Technologies (created with MS PowerPoint).

### 4.1 Sensors

MXenes are revolutionizing sensor technology in e-textiles through several groundbreaking mechanisms that significantly enhance performance compared to conventional materials. Their unique 2D layered structure provides an extraordinary surface-to-volume ratio (≈1500 m^2^/g) and abundant active sites, enabling ultrasensitive detection of mechanical, chemical, and biological stimuli.^128–130^ For strain/pressure sensing, MXenes’ exceptional electrical conductivity and piezoresistive properties enable remarkable sensitivity (gauge factors >5000) with ultrafast response times (<10 ms), capable of detecting subtle physiological signals such as venous pulses or even vocal cord vibrations.^131–135^ The materials’ tunable interlayer spacing facilitates selective ion transport, making them ideal for electrochemical biosensors that can detect biomarkers in sweat at concentrations as low as 1 nM. Furthermore, MXenes’ hydrophilicity and surface functional groups (O, F, OH) enable strong interfacial bonding with textile substrates, ensuring stable performance even under mechanical deformation.^136^ Their work-function tunability (4.1–5.3 eV) enables optimization for specific sensing applications, while the plasmonic properties of certain MXene compositions enable optical sensing modalities.^137–139^ Unlike traditional metal-based sensors, MXene-integrated textiles retain flexibility and breathability while achieving superior sensitivity, often with self-powered capabilities when combined with energy-harvesting functions.

The above figure (Figure 4) illustrates a generic MXene-based flexible sensor designed for strain or pressure sensing applications. The sensor typically comprises three main layers. At the base is a textile substrate that serves as a flexible, breathable foundation, allowing comfortable integration with the human body. Above this, a layer of MXene nanosheets—commonly Ti_3_C_2_T*_x_* —is deposited or embedded onto the fabric.^140^ This MXene layer functions as the primary sensing element due to its excellent electrical conductivity, mechanical flexibility, and sensitivity to structural deformation. The topmost layer is a thin, flexible protective coating made of materials such as PDMS or TPU, which shields the sensor from environmental damage while maintaining its mechanical compliance.^140–142^ When mechanical stimuli such as stretching, bending, or pressing are applied, the structure of the MXene layer changes—either by altering the spacing between flakes or by compressing the conductive pathways—leading to a measurable change in electrical resistance.

**Figure 4:**
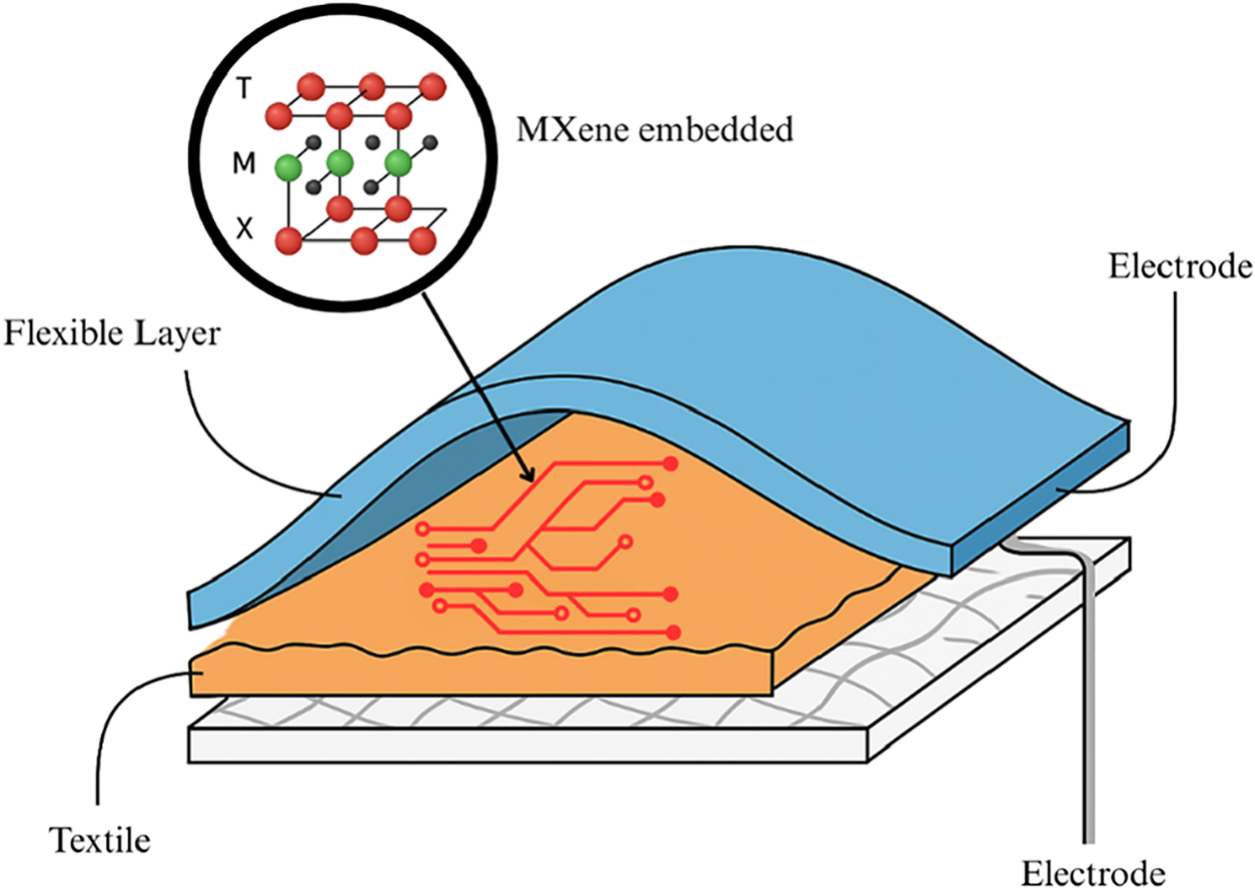
MXene-Based Flexible Sensor Architecture (created with MS PowerPoint).

One notable development involves the creation of a high-performance wearable strain sensor by depositing MXene nanosheets onto cotton fabric. This sensor achieved a gauge factor of 4.11 within a 15% strain range and demonstrated durability over 500 cycles, with a low strain detection limit of 0.3%. Such performance enables the detection of subtle human motions, including finger bending and eye blinking, highlighting its potential in health monitoring and motion detection.^143^ Further advancements include the integration of MXene with polyacrylonitrile nanofibers to form aerogels with three-dimensional porous structures. These composites exhibit enhanced mechanical properties and conductivity, contributing to the development of flexible pressure sensors with high sensitivity and rapid response times.^144^ Such innovations are crucial for applications in human–machine interfaces and soft robotics.^145^

Additionally, MXene-based pressure sensors have been engineered to achieve ultrahigh sensitivity at low pressures, enabling applications such as voiceless speech recognition and abnormal writing detection. These sensors demonstrate high sensitivity (*S* = 45.95 kPa^–1^ for pressures below 1 kPa), rapid response times of approximately 123 ms, and durability over 2000 cycles. This level of performance is particularly beneficial for assisting individuals with speech or writing impairments.^146^ This level of performance is invaluable for helping individuals with speech or writing impairments. Also, the integration of MXene into e-textiles has led to the development of personalized electronic textiles capable of ultrasensitive pressure sensing. For instance, combining MXene with PEDOT:PSS, which stands for poly­(3,4-ethylenedioxythiophene): polystyrenesulfonate, a widely used conductive polymer complex in the field of flexible and wearable electronics, including e-textiles and sensors, has resulted in biocompatible composites that maintain flexibility and demonstrate high sensitivity to pressure changes, making them suitable for wearable health monitoring systems.^147^

### 4.2 Energy Storage and Harvesting

Energy storage technologies are designed to store electrical energy for later use, providing a reliable, stable power supply on demand for various devices. Batteries, such as lithium-ion and sodium-ion batteries, store energy through electrochemical reactions, making them a popular choice for many applications.^148–150^ Alternatively, supercapacitors store charge physically at the interface between electrodes and electrolytes, utilizing electrostatic principles.^151,152^ These technologies play a vital role in ensuring consistent and dependable power for modern electronic systems. Energy harvesting, on the other hand, focuses on capturing ambient energy from sources such as mechanical motion, heat, and light and converting it into electricity.^153,154^ Triboelectric nanogenerators (TENGs) harness energy from friction or motion, while piezoelectric harvesters generate electricity from vibrations or pressure.^155,156^ Pyroelectric harvesters, meanwhile, capitalize on temperature fluctuations to produce voltage. The primary goal of energy harvesting is to enable self-powered systems that eliminate the need for batteries, offering a sustainable and innovative approach to powering devices.^157,158^

MXenes are revolutionizing energy storage in e-textiles through their exceptional pseudocapacitive behavior, achieving specific capacitances exceeding 2200 F/g in aqueous electrolytes—nearly triple that of graphene-based counterparts.^159,160^ Their accordion-like morphology creates a 3D ion transport network that enables ultrafast charging (80% capacity retention at 1000 mV/s) while maintaining mechanical flexibility, addressing the traditional trade-off between high energy density and textile deformability.^159,160^ The materials’ redox-active transition-metal cores (Ti, V, Mo) enable multielectron transfer reactions, thereby unlocking unprecedented charge-storage capabilities in flexible formats (Figure 5).^161,162^

**Figure 5:**
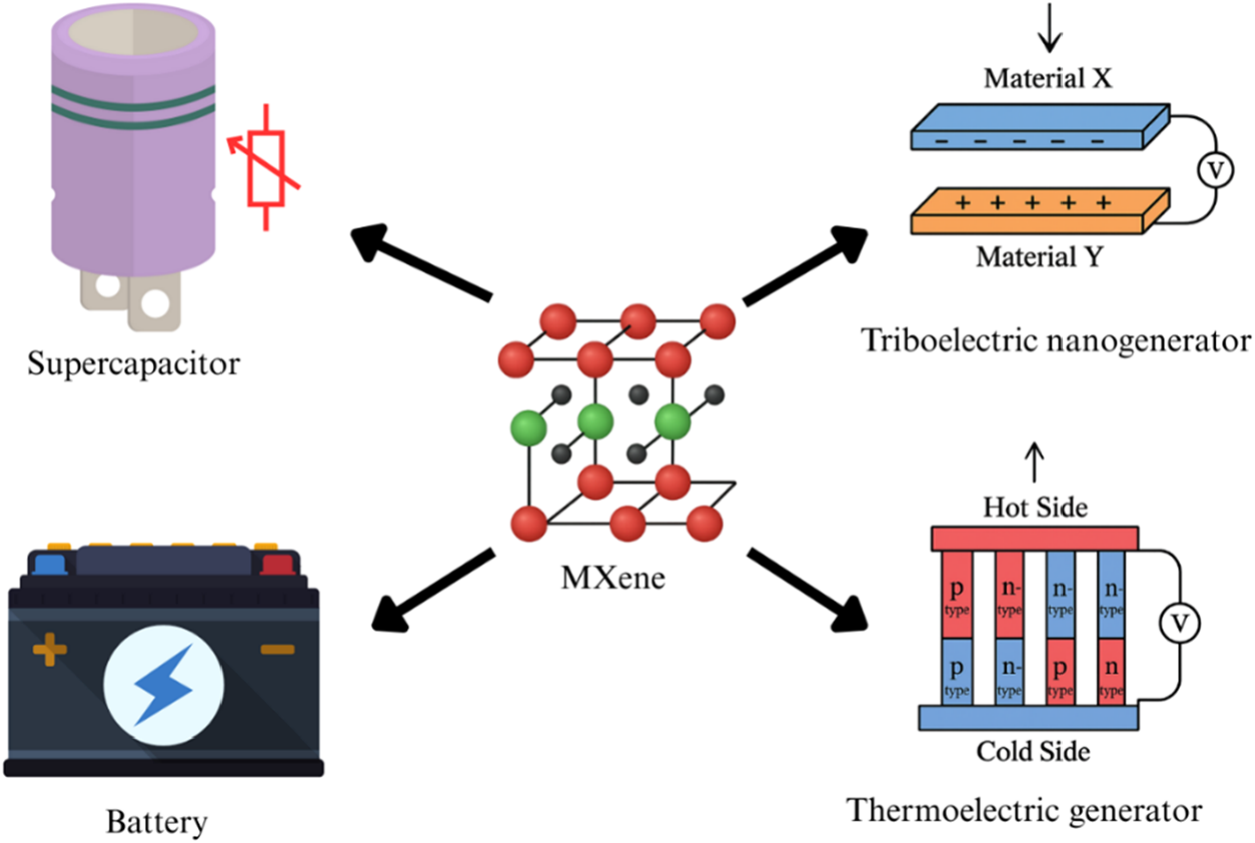
Dual Functionality of MXenes in Energy Technologies (created with MS PowerPoint).

For energy harvesting, MXenes’ intrinsic electronegativity and tunable work function (4.2–5.3 eV) optimize triboelectric output when paired with common textiles, generating power densities (15.6 mW/cm^2^) that surpass polymer-based systems.^163–165^ Their atomic thickness enhances contact electrification efficiency through quantum confinement effects, while surface terminal groups (−O, −F) create polarization gradients that boost piezoelectric responses by 300%.^166,167^ The materials’ anisotropic thermal conductivity (≈55 W/mK in-plane) simultaneously enables efficient pyroelectric conversion from body heat, with reported voltage outputs (1.2 V) sufficient for self-powered sensor operation.^168,169^ These dual energy-storage-harvesting capabilities are uniquely integrated into MXene textiles through solution-processable coatings that preserve the fabric’s hand feel (thickness <20 μm), overcoming the weight and rigidity limitations of conventional energy textiles.^170,171^ The synergy between MXenes’ charge storage kinetics and harvesting sensitivity is enabling autonomous e-textile systems that operate without external power sources, marking a paradigm shift in wearable energy technology. A comprehensive review explores how MXenes can be used to effectively harvest energy from mechanical, thermal, and solar sources. For instance, when used in TENGs, MXenes enhance charge transfer due to their high electrical conductivity and active surface functional groups. Similarly, in piezoelectric systems, combining MXenes with piezoelectric polymers improves stress transfer and increases electrical output. In thermoelectric devices, MXenes contribute to optimizing parameters such as the Seebeck coefficient (A coefficient that quantifies the voltage generated in a material due to a temperature difference, expressed in volts per kelvin, V/K), thereby improving the overall efficiency of thermal-to-electric energy conversion.^172^

On the energy storage front, MXenes have been widely investigated for use in supercapacitors and rechargeable batteries, including lithium-ion and sodium-ion batteries. Their layered structure allows efficient ion intercalation, while the metallic conductivity ensures fast electron transport. A recent study highlights the suitability of Nb_2_CT*_x_*, an MXene, for these applications. In lithium-ion batteries, MXene-based electrodes provide high specific capacities and excellent cycling stability. Moreover, their adjustable interlayer spacing makes them ideal for sodium-ion batteries, accommodating the larger Na^+^ ions without compromising structural integrity.^173^

### 4.3 Electromagnetic Interference (EMI) Shielding

Electromagnetic interference (EMI) shielding refers to the practice of attenuating unwanted electromagnetic radiation that can disrupt electronic device performance or cause harmful exposure in biological systems. EMI shielding materials are designed to reflect, absorb, or dissipate electromagnetic waves, ensuring signal integrity, data security, and compliance with regulatory standards (e.g., FCC, IEC). The key mechanisms of electromagnetic interference (EMI) shielding include reflection loss (SER), absorption loss (SEA), and multiple internal reflections (Figure 6). Reflection loss dominates in conductive materials such as metals and MXenes, arising from impedance mismatch between the shielding material and the incident electromagnetic waves. Absorption loss, on the other hand, occurs in materials with high dielectric or magnetic losses, where electromagnetic waves are attenuated through energy dissipation as heat. Multiple internal reflections are significant in porous or layered structures, where trapped electromagnetic waves undergo repeated interactions within the material, enhancing overall attenuation. Together, these mechanisms effectively minimize EMI.

**Figure 6:**
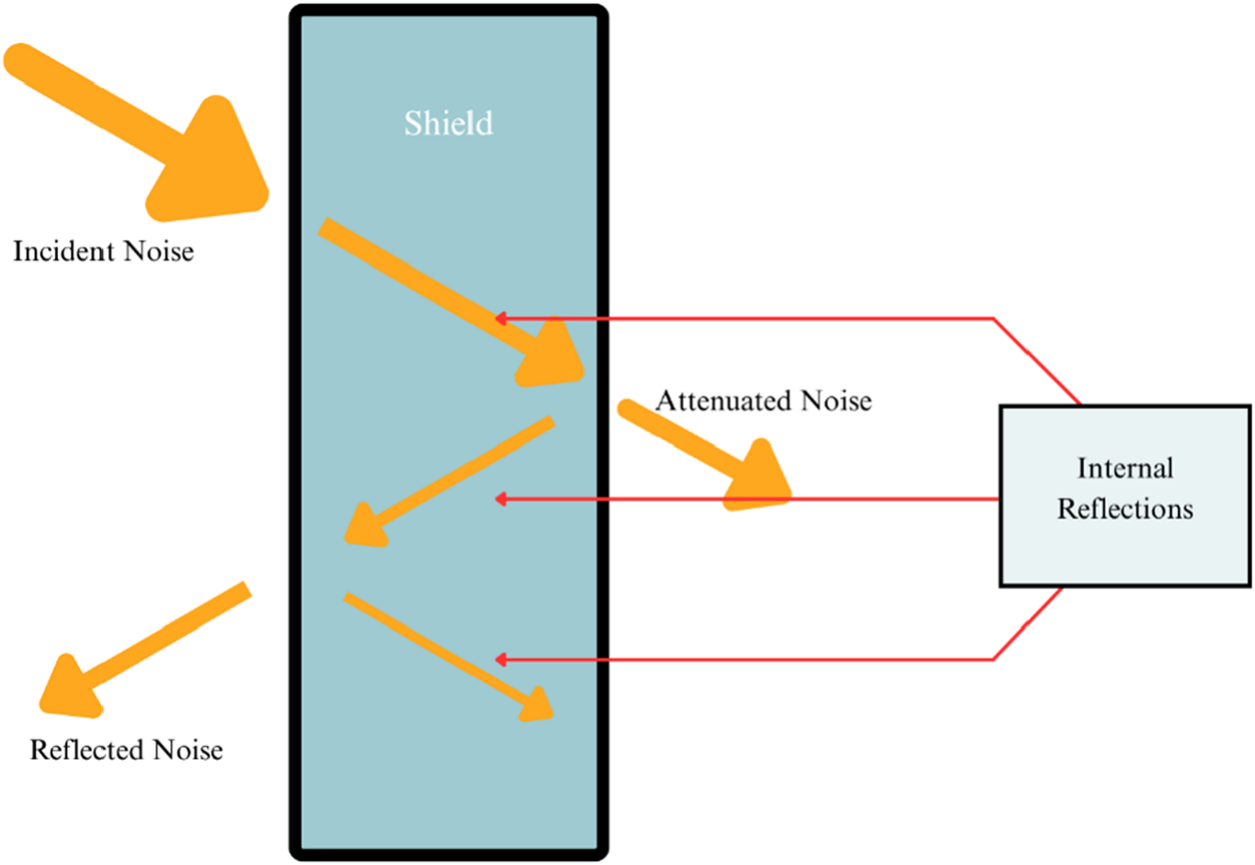
EMI Attenuation via Shielding Materials (created with MS PowerPoint).

MXenes have redefined performance benchmarks in EMI shielding, achieving >90 dB attenuation at thicknesses below 1 μm—far surpassing conventional materials such as copper (∼60 dB at 10 μm) or graphene-based shields (∼20 dB at 1 μm). This breakthrough stems from their metallic conductivity (∼10,000 S/cm) and layered morphology, which synergistically enhance wave reflection and internal scattering.^19^ For instance, a 45 nm-thick Ti__3__C__2__T*_x_* MXene film demonstrated 92 dB shielding effectiveness (SE), blocking 99.99% of incident radiation—a critical advance for miniaturized electronics.^174^

One of the primary advantages of MXenes in EMI shielding is their high electrical conductivity. To exemplify this, Ti_3_C_2_T*_x_* MXene films have demonstrated EMI shielding effectiveness (SE) values exceeding 90 dB in the X-band (8–12 GHz), outperforming many traditional materials. This performance is attributed to their metallic conductivity and surface functional groups, which contribute to polarization losses and enhance electromagnetic-wave attenuation.^119^ Beyond pure MXene films, researchers have developed MXene-based composites to enhance EMI shielding properties further.^175^ By incorporating MXenes into polymer matrices or combining them with other conductive fillers like carbon nanotubes or graphene, these composites achieve a balance between mechanical flexibility and shielding performance. Such composites not only maintain high SE values but also offer improved processability and mechanical strength, making them suitable for applications in flexible and wearable electronics.^119^

Research has also shown that MXene nanocomposites, due to their exceptional electrical conductivity, low density, and large specific surface area, are highly effective in absorbing and reflecting electromagnetic waves.^176,177^ This takes us to address electromagnetic interference (EMI) by effectively mitigating coupling mechanisms through their fundamental properties, as previously discussed. They have remarkable electrical conductivity, which enhances their ability to reflect and absorb electromagnetic waves, thus reducing radiation and conduction pathways (Figure 7). The diagram in Figure 7 illustrates the fundamental electromagnetic interference (EMI) coupling mechanisms, emphasizing pathways such as radiation, capacitive coupling, magnetic coupling, and conduction that transfer electromagnetic energy from a source to a receiver.^178^ Radiation entails energy propagation through free space, whereas capacitive and magnetic coupling involve interactions mediated by electric and magnetic fields, respectively. Conduction, on the other hand, relies on direct energy transfer through conductive mediums.^176,178,179^

**Figure 7:**
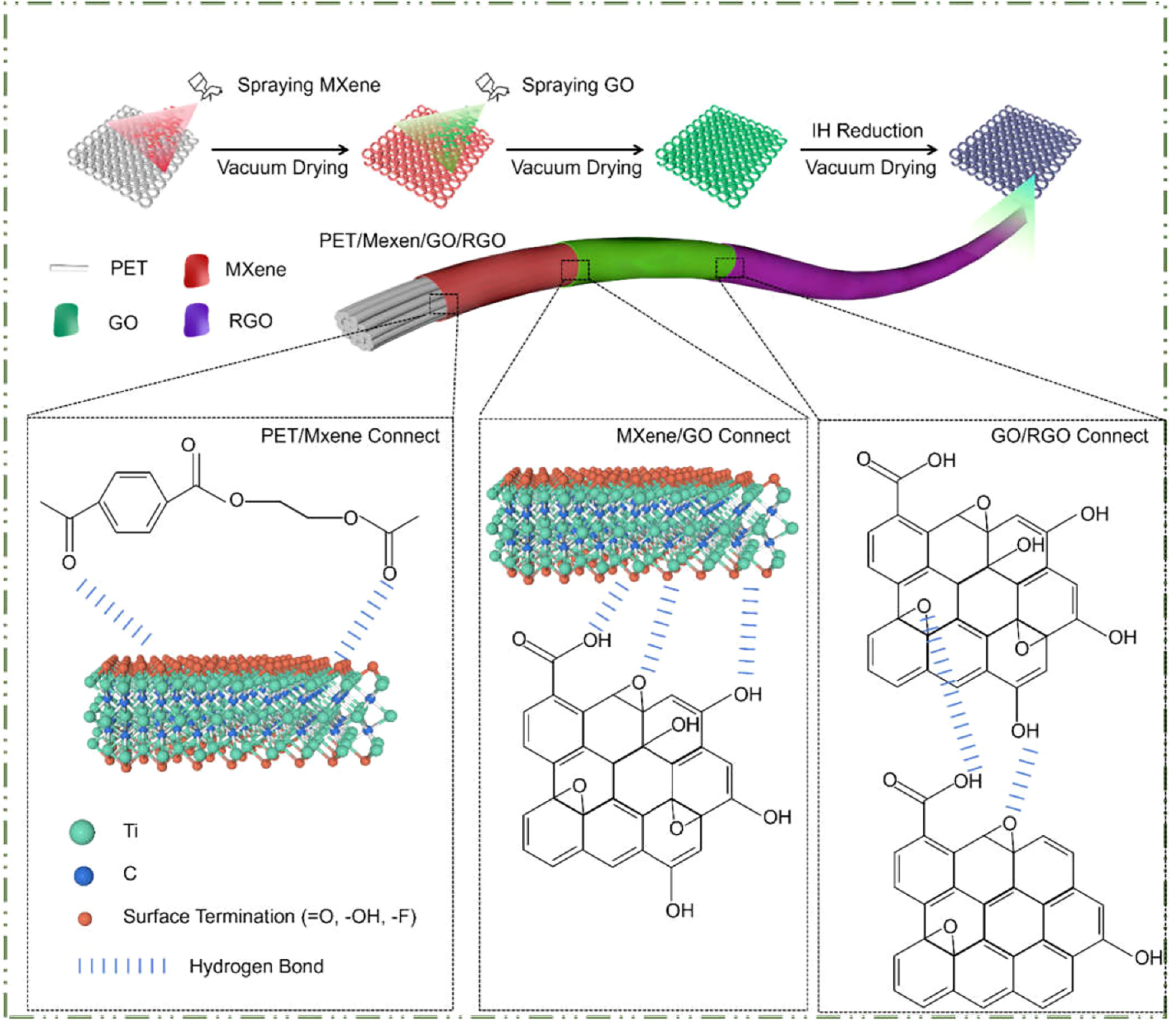
Preparation process and schematic diagram of the laminated mechanism for the PET/MXene/GO/rGO laminated flexible sensor. Reprinted with permission from ref 176. Copyright [2025] [MDPI].

On the other hand, recent developments in porous MXene structures, such as aerogels and foams, have opened new avenues for EMI shielding.^175^ These structures provide multiple internal reflections and scattering sites for electromagnetic waves, thereby enhancing absorption.^119^ For example, MXene-based aerogels have achieved an SE of around 61.4 dB, with a specific shielding effectiveness of 5155.46 dB·cm^3^/g, demonstrating their potential for lightweight, high-performance EMI shielding applications.^175^

### 4.4 Thermal Management

Thermal management in E-textiles refers to the integration of materials and electronic systems into fabrics that enable active or passive temperature control. This functionality is essential to enhance user comfort, safety, and performance across various applications. E-textiles designed for thermal regulation can generate or dissipate heat, monitor temperature changes in real time, and even respond autonomously to environmental or physiological conditions.^180^

A core mechanism for active heating in bright clothing is Joule heating—also known as resistive heating. This process converts electrical energy into thermal energy as electric current flows through a resistive material. When conductive fibers or coatings are embedded into textiles, they can produce controlled, localized heating based on Joule’s law: *Q = I* ^2^ *Rt*, where the heat (*Q*) generated depends on the current (*I*), resistance (*R*), and time (*t*).^181,182^ Conductive materials such as silver-coated yarns, carbon nanotubes, or stainless-steel threads are commonly used in such heating systems. These materials are flexible, lightweight, and can be integrated through weaving, embroidery, or screen printing.^183^

In contrast, cooling mechanisms often employ Phase Change Materials (PCMs), which absorb and release thermal energy at designated temperatures. For instance, PCMs can be microencapsulated and integrated into fibers or coatings; they absorb excess body heat and undergo a phase transition (e.g., from solid to liquid), storing the energy and later releasing it when temperatures drop.^184–186^ While thermoelectric cooling devices like Peltier elements can provide active cooling, they remain limited for textile integration due to bulk and power-consumption issues.^187,188^ Speaking of temperature, precision sensing is another critical component of thermal management. Standard sensors integrated into E-textiles include thermistors, RTDs (Resistive Temperature Detectors), and thermocouples, which can be woven or printed into fabrics. Fiber Bragg Grating (FBG) sensors, which use optical fibers to detect temperature variations along the fiber length, offer higher precision and are particularly useful in safety-critical applications.^180^ Often, these thermal components are managed by microcontrollers embedded within the textile system. They use sensor feedback to regulate heating or cooling output and may include wireless communication modules (e.g., Bluetooth, NFC) for transmitting data to mobile devices or monitoring systems.^183^ Some designs incorporate energy-harvesting mechanisms, such as solar panels or thermoelectric generators, to enable autonomous operation.

One significant advancement brought by MXenes is in passive radiative heating. Unlike conventional fabrics that emit body heat through mid-infrared radiation, MXene-coated textiles can minimize this heat loss. For example, researchers developed a monolayer Ti_3_C_2_T*_x_* MXene-coated polyester/polyurethane fabric that exhibited a significantly reduced mid-infrared emissivity of 19.53% in the 7–14 μm range. This design led to an increase in skin temperature by approximately 2.68 °C compared to standard cotton, without requiring any external energy input.^189^ This breakthrough highlights MXene’s potential in energy-saving thermal wear, especially for cold climates or outdoor environments. Also, MXenes have proven highly effective when integrated into active heating systems that use Joule heating. Liu et al. (2021) demonstrated a 3D stretchable textile combining MXene nanosheets and silver nanowires. The composite fabric exhibited excellent electrothermal performance, showing rapid and efficient heating when powered. This synergy between MXenes and metallic nanostructures not only improved heating response time but also maintained flexibility and stretchability, essential features for wearable textiles.^190^ In a different study, Janus fabric (which means textile has two distinct surfaces with different properties), well in this case, one side may be MXene incorporated, while the other side may be thermally insulating or breathable; found to achieve a 3.4 °C temperature increase in simulated skin by suppressing body radiation loss and achieving a 14.2 °C temperature increase under one sun irradiation.^191^ Also, to enhance oxidative stability and durability, MXene can be incorporated into advanced bright clothing by coassembling with natural sericin.^192^

### 4.5 Medical and Hygiene Textiles

The growing demand for textiles with antimicrobial properties, especially amid pandemic threats, has driven innovation in textile functionalization. Traditionally, antimicrobial properties in fabrics have been achieved through coatings of metal ions, plant extracts, and synthetic agents. However, concerns regarding environmental toxicity, leaching, and limited efficacy have led to a shift toward nanomaterials. Traditional antimicrobial textiles have primarily relied on metallic agents such as silver, zinc, and copper, which are effective at disrupting bacterial membranes and viral proteins.,^193^ as well as quaternary ammonium compounds (QACs) known for their broad-spectrum microbial action. Natural extracts, such as neem or aloe vera, have also been utilized for their biocompatibility, although their durability tends to be limited. However, these approaches face certain limitations, including leaching and a decline in efficacy after washing, potential toxicity to both skin and the environment, and weaker antiviral activity compared to their antibacterial performance.

Yu et al. (2024) introduced an innovative hybrid textile that integrates Zeolitic Imidazolate Framework-8 (ZIF-8) and MXene flakes into cellulose fibers, thereby significantly enhancing antibacterial and electromagnetic interference (EMI) shielding properties. This dual-functional textile not only effectively kills microbes but also provides EMI protection, making it particularly valuable for wearable electronics applications. The stability provided by ZIF-8, combined with MXene’s robust antimicrobial action through physical disruption and the generation of reactive oxygen species (ROS), underscores the material’s advanced capabilities.^194^ Building on MXene’s capabilities, Purbayanto et al. (2022) used interfacial engineering to coat MXene flakes onto polypropylene fabrics, highlighting the “nanoblade effect” for physical disruption of microbial membranes and reactive oxygen species (ROS) for chemical degradation. Additionally, the durability of these fabrics through washing cycles addressed a persistent limitation of traditional antimicrobial materials.^195^

On the other hand, Deng et al. (2023) advanced the field further by introducing MXene quantum dots (MQDs) into nanocoated cellulosic fabrics. Here, they not only achieved exceptional antibacterial efficiency but also featured biosensing capabilities for bacterial detection via fluorescence signals, demonstrating the potential of MXenes in next-generation medical garments.^196^ Sarac et al. (2025) expanded on these applications with a comprehensive review of MXenes in microbiology and virology, establishing them as versatile agents effective in both antimicrobial coatings and pathogen detection. The review underscored their eco-friendly and multifunctional potential, envisioning widespread applications across disciplines.^197^

Focusing on pandemic preparedness, Dwivedi et al. (2021) explored MXene–graphene composites, showcasing their synergy in antiviral and antibacterial action. This combination demonstrated promise for use in personal protective equipment (PPE) and public infrastructure, reinforcing MXenes as crucial materials for addressing future pandemics.^198^ Finally, Velidandi et al. (2024) integrated MXene nanosheets into silk textiles, balancing enhanced antibacterial properties with the softness and flexibility of silk. This work demonstrated the feasibility of antimicrobial wearables in luxury and consumer-friendly products, making MXenes accessible for everyday use.^199^

Traditional antibacterial systems, such as those relying on silver nanoparticles (AgNPs) or copper coatings, face significant limitations. These include short-term effects, such as metal ion depletion and cytotoxicity, leading to skin irritation caused by AgNPs.^200,201^ In contrast, MXenes demonstrate inherent antimicrobial activity, effectively eliminating over 99% of *Escherichia coli* and *Staphylococcus aureus* within 4 h.^197,199,202^ This is achieved through the physical disruption of bacterial membranes via their sharp edges, as well as oxidative stress induced by their surface terminations (−O, −F). Furthermore, MXenes exhibit long-term stability, maintaining their antibacterial efficacy even after 10 washes, unlike the leaching-dependent nature of AgNPs.^203–205^ Also, conventional masks and coverings like N95s usually compromise breathability for filtration, which may cause discomfort. In this case, MXenes offer a solution with electrostatic filtration, capturing 99.5% of aerosols while maintaining 85% air permeability.^206–209^ Additionally, photothermal MXenes, under NIR light, degrade pathogens, enabling reusable and comfortable protective gear.^210,211^

### 4.6 Communication Interfaces

The incorporation of MXene has significantly transformed communication interfaces by enhancing sensor performance, electromagnetic interference (EMI) shielding (as previously discussed), and human-machine interaction capabilities. MXene-based sensors exhibit remarkable electrical, electronic, and optical properties, making them highly effective for various sensing mechanisms, including electronic, electrochemical, and optical methods. These sensors are vital for gathering environmental data and transmitting it to data centers for informed decision-making, thereby improving communication interfaces in the era of the Internet of Things (IoT) with efficient real-time data exchange.^212^

The combination of MXene and graphene creates a robust framework that strengthens electromagnetic shielding and enhances thermal conductivity in polyolefin composites. This improvement is achieved through nanoscale interface engineering, including the formation of hydrogen bonds at the graphene/MXene interface.^213^ These advancements are critical for producing high-performance polymer composites used in microelectronics and microsystems, ensuring more reliable communication technologies.^214^ While discussing improvements to reliable communication strategy, it is also essential to consider responsiveness, as it increases the likelihood of achieving a high-quality, smooth connection in complex situations. For example, MXene-GaN van der Waals junctions in photodetectors deliver superior responsivity and drastically reduced dark current compared to traditional metal–semiconductor-metal photodetectors. These enhancements stem from the high-quality MXene-GaN interfaces, which optimize light extraction and photocurrent collection.^215^ Such breakthroughs make MXene-based photodetectors highly suitable for applications in underwater optical communication, paving the way for more advanced communication systems.

Admittedly, precision is as crucial as responsivity. Without the perfect balance of accuracy and responsiveness, the whole communication system will be suboptimal. In this case, an electroencephalogram (EEG) enables brain-machine interfaces (BMIs) or human-machine interfaces (HMIs), allowing individuals to control devices through brain signals.^216,217^ MXene-enabled self-adaptive hydrogel interfaces revolutionize active electroencephalogram (EEG) interactions by offering skin-compliant, motion-robust, and seamless human-machine interfaces. These advanced interfaces enhance signal transduction and deliver reliable electrical performance, allowing for high-precision detection of EEG signals.^218^ Such capabilities enable active control over human intentions, motions, and visual interactions, establishing new benchmarks for intuitive human-machine communication.^217^

MXenes are also known as a transformative material for advancing antenna technologies, particularly in applications requiring miniaturization. By achieving a thickness of 62 nm, MXenes have demonstrated the ability to support RF antenna functionality without sacrificing performance.^219^ This study is especially valuable for compact devices like Bluetooth-enabled wearables and flexible Internet of Things (IoT) devices. Speaking of wearables, printing MXenes directly onto flexible substrates has enabled the creation of RF resonators and sensors that seamlessly integrate with biological systems. This capability facilitates real-time wireless data transmission and opens up possibilities for wearable RF sensors.^220^ Moreover, MXenes have proven effective in creating multifunctional shielding composites. By combining MXenes with cellulose nanofibers, enhanced shielding from RF to IR wavelengths is achieved, which is crucial for integrated platforms where antennas and shielding layers must coexist.^221^

In the realm of fifth-generation (5G) communications, MXenes have shown significant advantages over traditional materials such as copper. These antennas exhibit reduced surface resistance and improved return loss, particularly in high-frequency mmWave and 5G bands.^222^ This improvement is driven by optimized interfacial charge transfer and reduced dielectric loss at the interface between MXene and its substrate, making them ideal for advanced wireless systems.^222^ Moreover, MXenes have been used to create low-profile sensor antennas for specialized VOC detection applications, while maintaining practical signal propagation across multimaterial interfaces. For wearable and skin-mounted devices, MXenes offer mechanical stability, preserving conductivity and ensuring robust performance even under deformation.^223^ These characteristics make MXenes indispensable for next-generation wireless technologies.

Beyond these applications, MXenes have enabled reconfigurable antenna designs through innovative approaches such as kirigami. Such designs allow frequency-response tuning via mechanical adjustments, offering a novel way to couple material geometry to electromagnetic behavior.^224^ Additionally, MXenes have proven valuable in cointegrating EMI shielding with antennas, effectively reducing reflection losses without sacrificing signal transmission—a breakthrough that eliminates the traditional trade-off between shielding and efficiency.^225^

## 5 Challenges and Limitations

### 5.1 Oxidation and Environmental Stability

Speaking of susceptibility to oxidation, MXenes, primarily composed of titanium carbide (Ti_3_C_2_T*_x_*) or other transition metal carbides, are highly susceptible to oxidation when exposed to air and moisture. This process significantly affects their electrical conductivity and mechanical integrity, as detailed in several studies.^226,227^ As MXenes degrade into oxides like titanium dioxide (TiO_2_), they lose their favorable conductive properties, which directly impacts the performance of MXene-based e-textiles, especially in applications that require continuous conductivity, such as sensors, actuators, and energy storage devices. On the other hand, humidity accelerates the oxidation of MXenes. Studies show that exposure to high relative humidity (RH) environments can increase degradation rates due to moisture absorption, thereby exacerbating oxide layer formation.^227^ This process compromises the long-term stability of MXene-based e-textiles, especially in wearable electronics, which are often exposed to sweat or rain. However, to combat the oxidation challenge, researchers are exploring surface functionalization techniques, such as coating MXenes with protective layers or integrating them with polymers. This approach helps shield MXenes from environmental exposure, thereby reducing oxidation rates and enhancing the durability of e-textiles.^228^ The integration of materials such as graphene oxide (GO) or silver nanowires (AgNWs) has been shown to provide protective effects, preventing oxidation while maintaining the required conductivity for e-textile applications.^190,229^

Enhancing the stability of MXene materials involves multiple strategies, including hybridization, surface modification, and cross-linking (Table 3). By combining MXene with sericin-modified carbon nanotubes, gallic acid, or polymers, protective networks or coatings are formed, significantly improving oxidative and environmental stability.^230–235^ Additionally, chemical treatments such as silylation or ionic liquid modification create hydrophobic or antioxidative layers that reduce oxidation rates and enhance long-term durability.^232,233,235,236^ Cross-linking MXene with polymers such as poly­(vinyl alcohol) or encapsulating it with hydrophobic polymers via vapor deposition further protects it against moisture and oxygen, ensuring sustained conductivity and robustness even under harsh conditions.^233–235,237^

**Table 3:** Approaches to Improve MXene Stability in E-Textiles

Approach	Effect on Stability	Citations
Hybrid networks (e.g., CNTs)	Enhanced oxidative stability	^237,239,240^
Antioxidant coatings (e.g., GA)	Improved wash/service durability	^240^
Silylation/Surface modification	Reduced oxidation, tunable surface	^232,233^
Polymer encapsulation	Long-term environmental stability	^234,235^
Ionic liquid modification	Quenches ROS, forms a protective cap	^236^

As previously discussed, the oxidation of MXenes can release metal oxides, such as titanium dioxide (TiO_2_), which are considered nontoxic in certain forms. Still, their nanoparticle form could pose environmental and health risks if they leach from e-textiles.^226^ The potential release of these materials into ecosystems, particularly through improper disposal or washing, raises concerns about their bioaccumulation and toxicity in aquatic environments. It has also been observed that the synthesis of MXenes often involves hydrofluoric acid (HF), which poses significant environmental and health risks due to its toxicity and corrosivity.^238^ This chemical challenge makes MXene production scalable for environmentally responsible manufacturing difficult. Although greener solvents or nontoxic etching methods can be utilized to reduce toxicity, these processes are still under development (Table 3).

### 5.2 Mechanical and Wash Durability

Mechanical durability in e-textiles is primarily assessed by subjecting the materials to conditions commonly encountered during wear and use, such as stretching, bending, twisting, abrasion, and physical deformation.^241,242^ These stresses can cause fatigue, cracking, or delamination of conductive pathways, thereby compromising electrical performance. Standardized test methods, such as the ASTM D4966 (Martindale Abrasion Tester) and ASTM D5034 (Grab Tensile Strength), are often employed to simulate wear and quantify mechanical resistance.^243,244^ In addition to these textile-based methods, cyclic mechanical testing under repeated strain—particularly for stretchable e-textiles—is performed to assess electromechanical stability, i.e., changes in electrical resistance as a function of cycles and deformation.^245,246^ A low drift in resistance values over multiple mechanical cycles is indicative of high mechanical durability.^245^ on the other hand, wash durability is intended for prolonged use under domestic or clinical laundering conditions. Washing introduces multiple stress factors, including water immersion, chemical exposure (detergents), thermal fluctuations, and mechanical agitation, all of which can affect the adhesion, continuity, and conductivity of integrated electronic components.^241,246^ The IEC 63302 and ISO 6330 standards have been proposed to address wash testing for smart textiles, incorporating specific washing protocols, temperature cycles, and detergent types.^247^ Further postwash assessments typically involve analyzing electrical performance using techniques such as the four-point probe method or multimeter resistance measurements.^248–250^ Additionally, a visual inspection helps identify potential issues such as delamination, cracking, or corrosion, while SEM or optical microscopy is utilized to examine surface morphology.^251^

MXene-based electronic textiles have evolved significantly from their early, fragile prototypes. Through coaxial fiber engineering, polymer composites, and material hybridization, multiple milestones have achieved substantial progress in mechanical resilience and wash stability. These embedded textile materials are strong, flexible, and durable, maintaining integrity through repeated bending and stretching. Cross-linking techniques and ultracompact fiber structures enhance strength and environmental resistance.^165^ Recent research has addressed the mechanical fragility and wash instability of MXene coatings in textiles by developing a robust multifunctional textile composite. Through thermo-chemo-mechanical optimization, mechanical durability and chemical resistance have been significantly enhanced, allowing for greater long-term stability. Studies have demonstrated that even after repeated deformation and exposure, the textile maintains effective electromagnetic interference (EMI) shielding and heating performance.^252^ Despite MXene’s natural degradation in wet environments, strategic polymer integration has been employed to mitigate this issue, ensuring sustained functionality in practical applications.^252^

Protective polymer coatings, cross-linking agents (e.g., borax, gallic acid), and hybridization (embedding) with other nanomaterials (e.g., carbon nanotubes, Ag nanoparticles) significantly improve the wash durability.^239,253–256^ These approaches strengthen the adhesion of MXene to textile fibers, preventing material loss during washing while maintaining conductivity and functional stability. Cross-linking agents, in particular, form strong intermolecular bonds that stabilize the MXene coating, reducing its susceptibility to degradation. For instance, when smart wearables are embedded with MXene, stable electrical conductivity and functional performance can be demonstrated after 10–50 washing cycles, with minimal increase in resistance and retention of antibacterial and sensing properties.^93,253–255,257^ This wash resilience is primarily attributed to the protective layers and reinforced bonding mechanisms introduced by polymer coatings and cross-linking agents. Therefore, the interface between MXene and the textile substrate is essential.^93,239,255^ Such bonding between MXene and textile fibers can be achieved through surface modification or microetching, preventing detachment of the conductive layer during washing. Not only does this approach increase the fiber’s surface roughness, but it also promotes better mechanical interlocking and improves MXene’s overall adhesion. In another study, a scalable fabrication method has been developed for coaxial fibers composed of MXene and multiwalled carbon nanotubes (MWCNTs) embedded within a thermoplastic polyurethane (TPU) matrix.^258^ These fibers exhibit exceptional stretchability and durability, making them particularly well-suited for human motion detection. Findings indicate that the fibers maintain their mechanical integrity even under cyclic stretching and bending, and that signal reliability remains after 50 washing cycles.^258^ Thus, the combination of MXene’s conductivity and MWCNT’s structural toughness has resulted in a hybrid textile capable of long-term sensing applications. Additionally, the material demonstrates excellent mechanical recovery and fatigue resistance under dynamic-body-motion simulations, reinforcing its potential for wearable technology.^258^

On a different note, flake detachment and cracking of MXene layers are very significant degradation mechanisms, particularly under cyclic mechanical stress (bending, stretching) and washing. This problem undermines the conductivity, mechanical adhesion, and sensor performance of MXene-integrated textiles, limiting their practical use in wearables. Repeated mechanical stress often leads to interfacial delamination and cracking between MXene layers and the substrate, compromising durability. Studies have shown that after extensive fatigue-bending tests, cracks form in MXene-coated structures without polymer reinforcement. To mitigate this issue, a nonadditive polymer coating has been employed, effectively reducing flake detachment while providing self-healing under minor stress.^259^

Flake detachment during water exposure and flexural fatigue stems from poor adhesion between MXene and textile fibers, edge degradation due to oxidation, and structural stress concentrations around wrinkles. To counteract these vulnerabilities, encapsulation strategies have been recommended, which significantly reduce delamination and help maintain conductivity even after 50 or more washing cycles.^260^ But when compared to GO/AgNW coatings, MXene exhibited lower recovery from crack propagation. To address this limitation, cross-linking interlayers and hybrid flakes have been proposed to prevent progressive cracking and ensure long-term mechanical stability under dynamic usage conditions.^261^

Oxidation at flake edges is another critical challenge, as it weakens interflake bonding and increases the likelihood of peeling. Studies show that after 100 fatigue cycles, unreinforced MXene structures experience a substantial loss in conductivity. However, encapsulation and polymer blending techniques have been effective in extending performance beyond 300 cycles, preventing detachment and maintaining material integrity.^251^

### 5.3 Cytotoxicity and Biocompatibility

Cytotoxicity refers to the ability of a material or substance to induce cell damage or death. In the context of e-textiles, cytotoxicity assessments are necessary to determine whether the conductive components, such as metal nanoparticles (e.g., silver, copper), conductive polymers (e.g., polypyrrole, PEDOT:PSS), or carbon-based nanomaterials (e.g., graphene, carbon nanotubes), exhibit harmful effects on dermal or epithelial cells.^241,262,263^ Leaching of these materials from textile substrates, either due to perspiration, mechanical abrasion, or environmental exposure, poses a potential risk of skin irritation or cellular toxicity.^241^ Standard cytotoxicity tests, such as the MTT assay, lactate dehydrogenase (LDH) release assay, and live/dead cell staining, are commonly used to quantify cellular viability in the presence of textile extracts or residues.^264–266^

In parallel, biocompatibility is a broader term encompassing the overall compatibility of a material with biological systems, particularly its ability to perform desired functions without eliciting undesirable local or systemic effects.^241^ For e-textiles, biocompatibility assessments involve evaluating not only the absence of cytotoxicity but also ensuring that the material does not provoke immune responses, allergic reactions, or chronic inflammation upon prolonged skin contact.^241^ The interaction of conductive coatings, binders, and functional finishes with the epidermis must be benign, especially in applications such as electrocardiography (ECG) garments, wound monitoring textiles, and wearable biosensors.^267,268^ In vivo studies, patch testing, and skin irritation assays are typically employed to establish the biocompatibility profile of such materials.^242,269,270^

Admittedly, MXene-based electronic textiles (e-textiles) have emerged as promising candidates for next-generation wearable electronics due to their superior conductivity, flexibility, and responsiveness. However, the transition from material innovation to biomedical application necessitates thorough evaluation of their cytotoxicity and biocompatibility, particularly given their intended direct contact with the skin or underlying tissues. This is a crucial step to ensure biosafety in applications such as physiological monitoring, wound care, and epidermal diagnostics.

In vitro studies have demonstrated that specific MXene compositions, such as Ti_3_C_2_T*_x_*, exhibit generally low cytotoxicity across various mammalian cell lines. These findings have positioned MXenes as viable candidates for skin-interfacing technologies. However, their biocompatibility is not universal and depends on several intrinsic and extrinsic factors. Surface terminations (e.g., −F, −OH, −O), degree of oxidation, flake lateral size, and concentration all play determining roles in the cellular response.^271^ For instance, flake sizes below 100 nm, while desirable for high surface area and reactivity, have been associated with increased cellular internalization and subsequent oxidative stress.^271^

Further, evidence from metabolic activity assays (MTT and CCK-8) in fibroblast and epithelial cell lines indicates that MXene dispersion concentrations exceeding 200 μg/mL can suppress cellular proliferation and viability. However, this effect can be mitigated mainly through surface functionalization.^272,273^ Techniques such as PEGylation, PVA coating, or incorporation of biopolymers like gelatin and silk fibroin have shown considerable promise in reducing cytotoxicity.^273^ These modifications act as physical and chemical barriers, limiting direct cell–MXene interaction and thereby preserving cellular integrity.

Biocompatibility assessments extended to composite structures have demonstrated that MXene integrated into porous or elastomeric matrices—such as polyurethane or polydimethylsiloxane—can maintain high levels of cellular compatibility. One particularly effective strategy involves embedding MXene flakes in porous textiles or sponges, enhancing oxygen permeability and skin conformity while reducing local stress points. These structures not only improve the mechanical comfort and breathability of wearable devices but also mitigate potential cytotoxic responses by keeping active flakes away from dermal contact.^274^

Concentration- and time-dependent effects also play a role in biocompatibility. Prolonged exposure (>48 h) at moderate concentrations has not shown significant adverse effects in some studies; however, chronic exposure scenarios remain underexplored. While most in vitro studies report high biocompatibility within the 24–72-h window, standardized protocols aligned with ISO or OECD guidelines are urgently needed to harmonize testing and enable comparative risk assessments across laboratories.^271^ On the other hand, biocompatible coatings, especially those based on hydrogels and natural polymers, serve dual roles—enhancing the material’s mechanical durability and preventing MXene oxidation, which can generate harmful byproducts. In cases where MXenes are combined with other conductive fillers (e.g., PEDOT:PSS or graphene derivatives), synergistic improvements in both performance and safety have been observed.^275^

Importantly, early stage wound-monitoring devices using MXene composites have demonstrated high cellular viability in keratinocytes and fibroblasts, suggesting their potential in real-time medical diagnostics. These results, while promising, underline the importance of controlling flake dispersion, anchoring strategies, and avoiding long-term direct exposure in physiological conditions without protective layers.^275^

### 5.4 Environmental and Lifecycle Concerns

The incorporation of MXenes into wearable electronic textiles, depicted in Figure 8, raises significant environmental and lifecycle concerns. One of the most pressing issues is the unknown long-term effects of MXene materials on human health and the environment. Unlike conventional textiles, MXenes are two-dimensional materials with unique electrical and mechanical properties, but their interactions with biological systems and ecosystems remain poorly understood. MXenes, a novel family of two-dimensional layered materials, are carbides, carbonitrides, borides, or transition metal nitrides that are created by selective etching. MXenes are potential adsorbents that have been investigated for a variety of pollutants due to their high surface area, activity, biological compatibility, and chemical stability.^276^ Though the potential for leaching of MXene compounds during washing or wear could pose risks, particularly if these materials are found to be toxic or persistent in the environment.^276^ Furthermore, established disposal methods for MXene-embedded textiles have not yet been developed. Waste management frameworks for recycling or safe disposal are crucial, as MXenes may not be biodegradable and could contribute to environmental pollution if not appropriately handled. Although a more systematic study is required to fully understand the biosafety concerns and biological impacts of MXenes and MOFs before clinical trials, the number of studies examining their toxicity and biocompatibility is increasing. Strong acids and high temperatures are frequently used in the synthesis of MXenes, which might have negative environmental implications if improperly handled. Throughout the production process, steps should be taken to guarantee appropriate waste management and reduce the emission of hazardous byproducts. Assessing the possible environmental release of MXenes after their use in biomedical applications is also essential. There are various obstacles to the biological uses of MOFs. The search for stable and water-resistant MOFs faces several challenges, including high fabrication costs, low capacity, poor selectivity, and difficulties with recycling, regeneration, and preserving chemical, thermal, and mechanical stability.^276^ Consequently, the growing production and environmental usage of MXene may lead to its unavoidable discharge into the environment, thereby harming both the ecosystem and human health. The evaluation of MXene’s environmental destiny and risk, as well as its ecological application, depends heavily on our understanding of its dispersion, aggregation, and oxidation stability. Furthermore, the academic community is particularly concerned about the safe use and biological effects of MXene given its widespread use.^276^ Therefore, addressing these lifecycle concerns is vital to ensure the sustainability of wearable electronic textiles that utilize MXenes.

**Figure 8:**
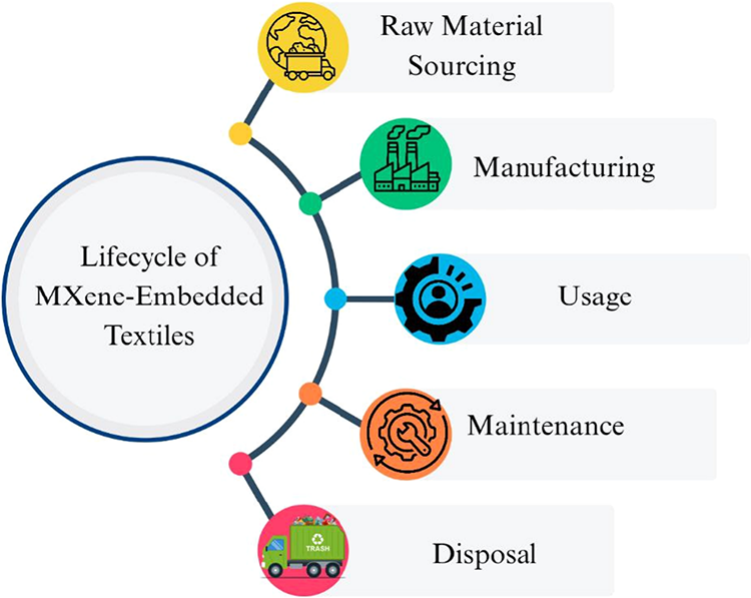
Lifecycle of MXene-Embedded Textiles (created with MS PowerPoint).

### 5.5 Manufacturing Scalability

Manufacturing scalability is another significant barrier to the widespread adoption of MXenes in wearable electronic textiles. Restacking limits the use of MXenes, as it does for other 2D compounds. However, by forming composites, additives can act as a separator between MXene layers, preventing restacking.^276^ While academic research has demonstrated promising applications of MXenes in lab settings, translating these findings into mass production involves complex challenges. The transition metal carbides, nitrides, and carbonitrides that make up MXenes, the possibly largest class of 2D materials, offer exceptional practical qualities that could find utility in energy technology, communication, and several other areas. They have the potential to go from laboratory use to more extensive industrial manufacturing because they are made utilizing a scalable, selective etching technique. According to Figure 9, key factors include ink formulation, which must ensure that MXenes remain stable and well-dispersed in printing inks to maintain their functional properties. When it comes to easily printable functional inks for energy-storage devices, two-dimensional materials are appealing options.^276^ Moreover, the drying behavior of MXenes can affect adhesion and conductivity, necessitating optimized drying processes to prevent cracking or separation during production.^276^ Additionally, industrial processing gaps exist; current techniques may not be readily suitable for large-scale applications. For example, techniques such as screen printing or inkjet printing need to be adapted to consistently apply MXenes onto various textile substrates while maintaining high-quality performance.^276^ Overcoming these scalability hurdles is essential for integrating MXenes into commercially viable wearable electronic textiles.

**Figure 9:**
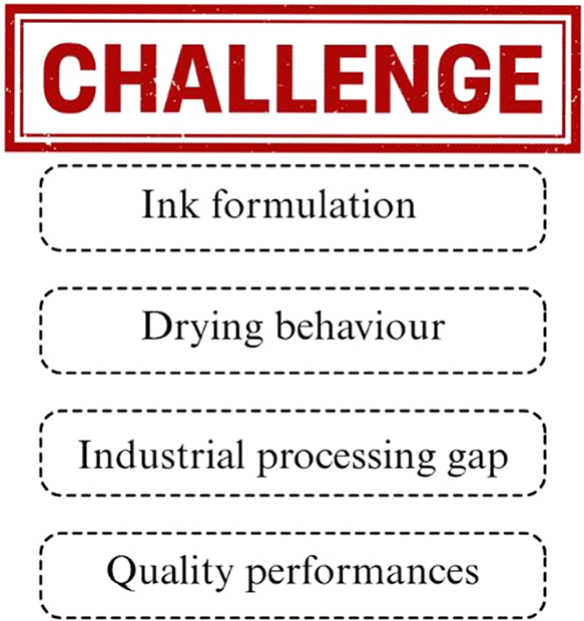
Challenges in Scaling MXenes for Wearable Electronic Textiles (created with MS PowerPoint).

The primary process for producing MXenes is the selective etching of MAX phases, which can be scaled up using fluidized-bed reactors, electrochemical etching, or molten-salt etching. To ensure consistent quality, purity, and yield across large batches, these methods must be optimized.^276^ Variability makes large-scale repeatability more challenging, while the variety of MXene structures and compositions enables customization for specific purposes. Controlling characteristics such as defect levels, surface terminations, and layer thickness requires standardized synthesis processes. Performance is limited by restacking, which decreases surface area and electrical conductivity. To sustain exfoliation and improve electrical conductivity, spacers such as organic molecules, polymers, or other nanomaterials (e.g., graphene or carbon nanotubes) can be added. MXenes must stay stable and evenly distributed to create functional inks. By altering the surface using surfactants or polymer brushes, colloidal stability can be increased, avoiding aggregation during printing and storage.^276^ For printing techniques like screen printing or inkjet printing, ink compositions should possess appropriate rheological characteristics. Printability and film formation are affected by changes to the solvent composition, binder content, and additives. Specialization in MXene inks is necessary for processes such as screen printing, aerosol jet printing, and roll-to-roll processing to ensure accurate deposition without clogging nozzles or damaging textiles.^276^

Following MXene modification, printing inks provide strong textile integration and unique sensing benefits for smart wearables, such as customized rheology and enhanced adhesion for consistent, comfortable deposits; high conductivity, large surface area, and tunable surface chemistry for quick transduction of strain, temperature, pH, gases, and electrochemical signals; compatibility with inkjet, screen, and gravure printing for scalable, accurate patterning on complex textiles; improved durability under wear conditions like flexing, washing, and abrasion; and quick integration from synthesis to sensor-enabled textiles with streamlined workflows. Optimizing ink viscosity and drying on various textiles, functionalizing MXene for improved fiber bonding and targeted sensing, safeguarding sensors with breathable encapsulation, and evaluating biocompatibility and safety while coordinating power, processing, and wireless integration should be future priorities.^71,260,277^ Following the correct drying procedures is essential. Cracking and delamination can be avoided with carefully regulated humidity and temperature. Mild annealing is one post-treatment procedure that may improve conductivity and adhesion without degrading the textile substrate. It is crucial to create formulations that work with flexible and washable textile surfaces. Encapsulation layers can protect MXene coatings from environmental deterioration and washing cycles.^276^ Energy use and hazardous waste should be reduced in large-scale production. Manufacturing can become more sustainable by investigating solvent-free techniques or more environmentally friendly etching chemicals. Safe handling practices and proper storage conditions are essential during manufacturing, as MXenes are sensitive to oxidation and environmental pollutants.^276^ The use of cheap raw materials, process automation, and economies of scale can reduce production costs. Strict quality assurance procedures are implemented to ensure consistency in MXene characteristics, which is essential for commercial applications. By bridging the gap between academia and industry, obstacles in scalable manufacturing and application development can be addressed, and technology transfer accelerated.^276^ However, a great deal of work still needs to be done on more varied MXene structures and compositions, processing optimization, and the link between synthesis, structure, and property. Before doing applied research, the larger scientific community must take its viability into account.^276^

### 5.6 Lack of Standards

The lack of uniform testing standards for MXene-integrated textiles poses a significant challenge in validating their performance in wearable applications. Currently, no established framework provides standardized methods for assessing key parameters, such as conductivity, flexibility, and wash fastness, of these materials. This absence of standards complicates comparisons across different studies and hinders confidence in the market.^276^ For instance, the lack of standardized conductivity tests may result in inconsistent performance expectations between products, leading to consumer dissatisfaction. Similarly, without standardized wash fastness testing, the durability and practical usability of MXene-equipped textiles remain uncertain, which can deter potential adoption by textile manufacturers.^276^

Figure 10 elaborates that research, development, and commercialization are all impacted by the lack of standardized testing procedures for textiles containing MXene. Properties such as conductivity, flexibility, and wash fastness may be measured differently across producers and laboratories in the absence of standardized protocols. It is challenging to evaluate outcomes or determine actual performance because of this discrepancy.^276^ Manufacturers may offer performance information derived from internal testing, which is not always comparable or widely accepted. This may result in overstated claims, customer deception, and doubts about the system’s reliability. MXene-based textiles must meet specific performance requirements verified by accepted standards to be widely adopted, particularly in vital applications such as military equipment and health monitoring.^276^

**Figure 10:**
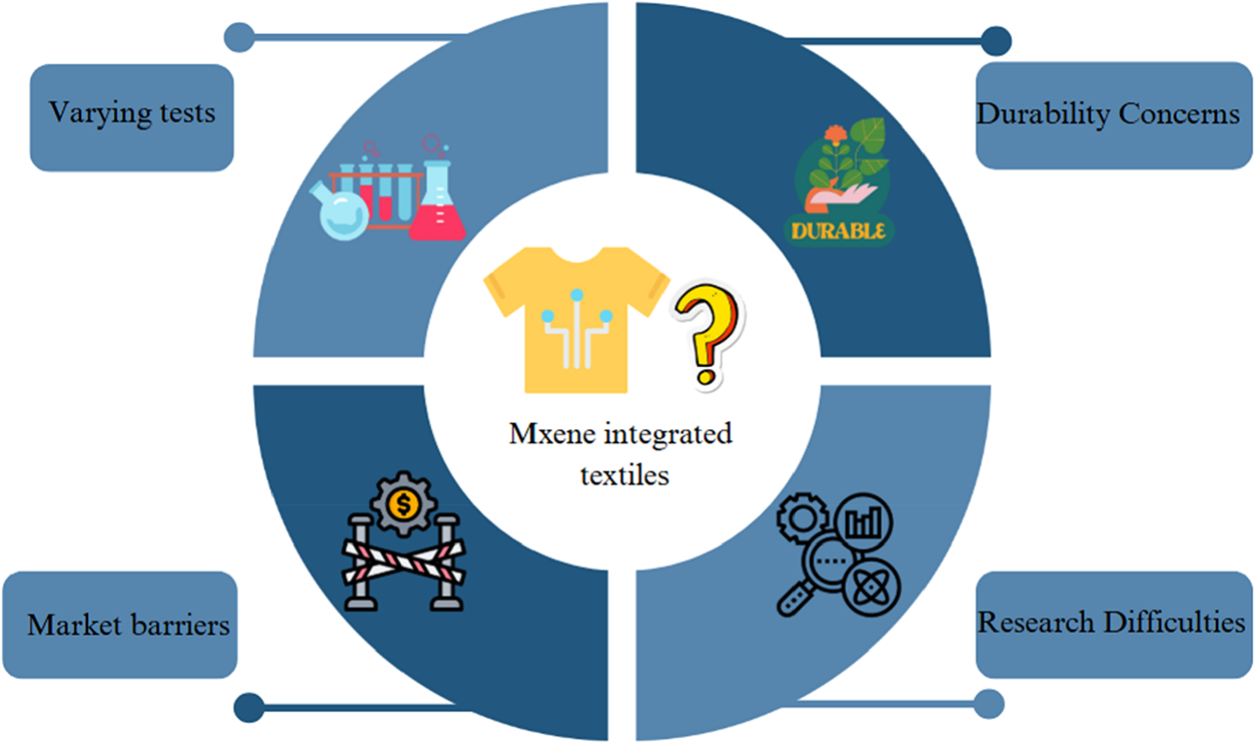
Challenge: No Standards for MXene-Integrated Textiles (created with MS PowerPoint).

Regulatory approval is delayed, and the absence of such standards hampers certification procedures. The ability of textiles to retain their electrical and functional properties after laundering is assessed through wash fastness testing. Durability promises are subjective in the absence of standardized testing, leaving customers at risk of dissatisfaction if the textiles break down too soon.^276^ Standard procedures are used in scientific research to guarantee reproducibility. Conflicting data from different testing techniques might impede the understanding of material characteristics and limitations in practical applications.

Temperature, humidity, and contact resistance are all important considerations for measuring electrical conductivity in textiles. Test configurations, measuring electrodes, and environmental variables should all be specified in standard operating procedures. Standardized mechanical testing procedures, such as those for bending, stretching, and cyclic deformation, should be followed when conducting quantitative tests of stretchability and flexibility. To determine retention of qualities after several washes, tests should mimic actual laundry settings, including the washing temperature, the chemicals used, and the mechanical agitation.^276^ Researchers, textile producers, regulatory organizations, and standardization organizations such as ISO or ASTM work together to develop industry-wide standards. Industries and consumers can therefore trust performance claims. Well-defined standards facilitate regulatory approval and certification. Researchers can advance innovation by building on similar data. Standardized performance standards foster industry expansion and increased consumer confidence.^276^

## 6 Industrial and Commercial Outlook

### 6.1 Market Growth and Demand

According to Figure 11, the market for smart textiles is witnessing significant expansion, particularly in sectors such as healthcare and wearable technology. Smart textiles are increasingly being used in garments that monitor vital signs such as heart rate and temperature in real time, facilitating at-home healthcare. Examples include bright shirts that can send alerts to healthcare providers based on the data received. MXenes are essential for the development of neural interfaces and biosensors due to their exceptional conductivity and compatibility with biological systems. Their practical signal transduction skills improve the field’s potential for diagnosis and treatment by enabling accurate monitoring of physiological processes. Additionally, MXenes are essential in tissue engineering, where their unique blend of biocompatibility and mechanical strength enables scaffold materials that promote cell adhesion, proliferation, and differentiation. Smart bandages are designed to monitor wound conditions (e.g., infection) and can administer medication as needed. Innovations in textiles enable embedded sensors that provide caregivers with feedback on the state of the wound.^276^

**Figure 11:**
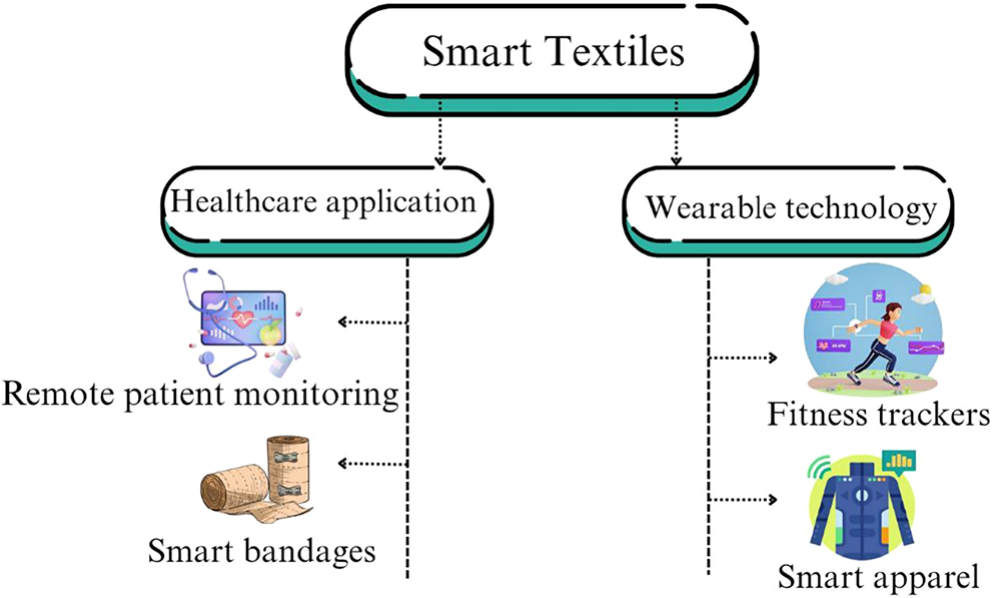
Significant expansions of smart textiles (created with MS PowerPoint).

Textile-based sensor devices, including patches, contact lenses, and forgings, have attracted greater attention in the tissue engineering community in recent years. For example, Xu et al. created a skin patch that uses ultrasonic pulses to track the wearer’s heart rate and blood pressure.^276^ For the early detection of cardiovascular issues, this information is essential. Devices integrating smart textiles to track users’ physical activity, sleep patterns, and heart rates are gaining traction. Clothing equipped with technology, such as temperature regulation, moisture-wicking, and monitoring capabilities, is becoming popular among fitness enthusiasts and outdoor adventurers..^276^

Shifting consumer preferences are driving demand for health-centric wearables and intelligent textiles. Consumers are increasingly aware of their health metrics, driven partly by the wealth of health data available through apps and devices. The rise of telehealth services during the COVID-19 pandemic has further popularized health-focused textiles. Individuals aspire to seamlessly integrate technology into their daily lives, leading to greater acceptance of smart textiles. The convenience of monitoring health through everyday clothing without the need for additional devices attracts tech-savvy consumers. Bright garments that combine professional attire with wearable technology cater to the modern consumer who desires functionality such as breathability and ease of care without sacrificing style. Regulatory frameworks have a vital role in shaping the smart textiles market.^278–281^

For wearables intended for health monitoring, the FDA provides specific guidelines to ensure the safety and efficacy of these devices before market release, fostering consumer trust. Organizations such as ISO (International Organization for Standardization) set standards for smart textiles, particularly related to biocompatibility and environmental impact, influencing manufacturing practices in the industry. Increasing regulations aimed at reducing waste in the textile industry, including the push for circular-economy practices, are spurring innovation in the use of sustainable materials in smart textiles.^282–285^

### 6.2 Patents and Prototypes

MXenes, a family of two-dimensional transition metal carbides and nitrides, exhibit unique properties, including high electrical conductivity, mechanical strength, and chemical stability. Figure 12 presents their incorporation into textiles opens up a range of innovative applications, particularly in healthcare and smart technology. Moreover, MXenes are a novel class of two-dimensional (2D) transition-metal carbonitrides or carbides that resemble graphene in structure. M_*n*+1_X*_n_* T_*x*_ is the generic formula for MXenes, where M is an early transition metal element, X stands for carbon, nitrogen, and boron, and T is a group that contains fluorine or oxygen on the surface.^282–285^ These new 2D materials have a large specific surface area, high conductivity, and stability, as well as unique 2D layered structures. Because of these characteristics, MXenes have attracted more attention and have become new substrate materials for investigating applications in medicine delivery, environmental adsorption, energy conversion and storage, photothermal treatment, and catalytic degradation.^282–285^

**Figure 12:**
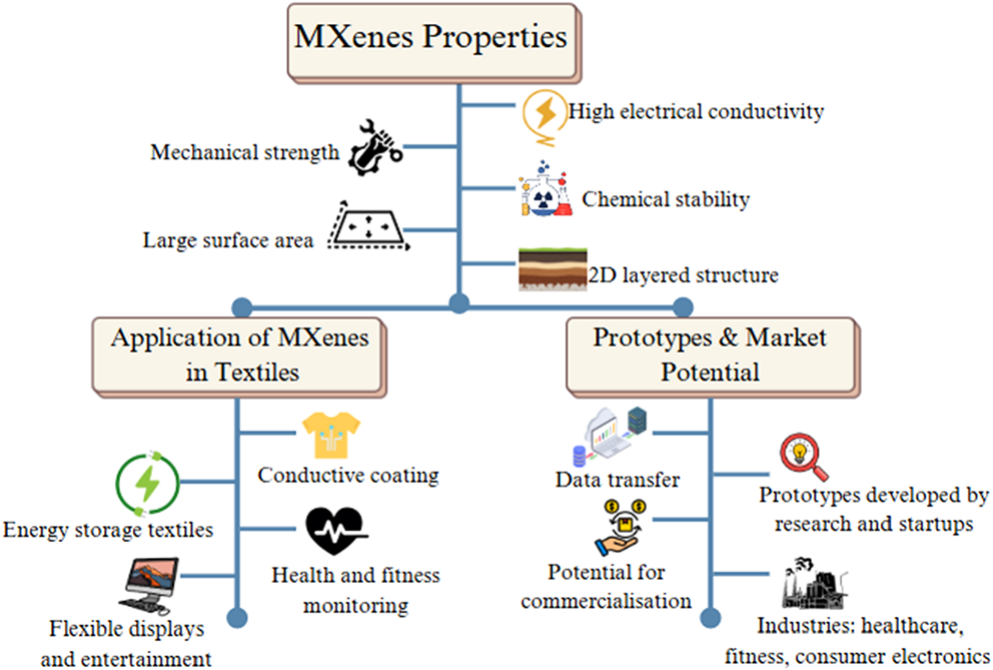
MXenes in Smart Textiles: From Properties to Applications (created with MS PowerPoint).

One significant area is the development of textile coatings using MXenes to enhance electrical conductivity, enabling fabrics to function in wearable electronics. Patents also cover MXene-integrated textiles capable of energy storage. Several research institutions and startups have made progress in developing prototypes incorporating MXenes. For example, Smart shirts for health monitoring: technology has created a prototype bright shirt embedded with MXene sensors that track heart rate and body temperature, sending data to a connected smartphone app. Moreover, flexible displays have enabled the creation of LED displays woven into fabrics, showcasing video and graphics for entertainment or information dissemination.^282–285^

The surge in patents and prototypes signifies not only a robust academic interest but also the potential for commercialization in diverse fields such as healthcare, fitness, and consumer electronics. The innovations revealed by these patents could lead to the proliferation of textiles with functionalities beyond traditional fabrics, thereby meeting the growing market demand for integrated technology in everyday clothing.^282–285^

### 6.3 Technology Readiness and Barriers

It is essential to recognize that, despite notable progress, many MXene applications for textiles are still in the research and development (R&D) stage, as assessed by the Technology Readiness Level (TRL). Knowing the TRL spectrum makes it easier to predict when these technologies will become useful and start to attract customers. Level of Present Technology Readiness: Early to midstage development is still the state of most MXene technologies.^282–285^ Although fundamental scientific concepts, including the conductivity and stability of MXenes, have been proven in TRL 2 (Concept Formulation), textile applications are still purely theoretical. Laboratory prototypes, such as MXene-coated textiles or textile sensors integrated into textiles, have been developed for TRL 3–4 (Proof-of-Concept & Validation), as shown in Figure 13. Though they lack comprehensive real-world testing, durability evaluations, and scaling, some prototypes show promise.^282–285^ The Path to Higher TRLs (5 and beyond) for MXene requires testing in real-world scenarios, such as long-term durability, environmental exposure, washing, and wear. Transferring from lab-based synthesis to pilot-scale production while preserving quality and fulfilling performance, safety, and environmental requirements to make market access easier. Some obstacles to widespread adoption include the high cost of manufacturing, etching, delamination, and surface functionalization are some of the multistep procedures used to produce high-quality MXenes.^282–285^ These procedures frequently call for costly equipment, exact controls, and dangerous materials. Costs can rise dramatically when laboratory procedures are scaled up to an industrial level. The high costs of materials and processes hamper cost-competitive textile production. Furthermore, maintaining consistent qualities (such as surface chemistry and electrical conductivity) across several production batches remains challenging.^282–285^ Inconsistent sensor sensitivity, electrical performance, or textile durability might result from variations in MXene quality, which undermines dependability and confidence. Another is Long-Term Stability: MXenes are susceptible to environmental degradation and oxidation, particularly in hot or humid conditions. The functional layers may deteriorate over time due to exposure to heat, moisture, washing, and mechanical forces, thereby reducing the effective lifespan and user confidence.^282–285^ Furthermore, Market Acceptance and consumer education regarding the advantages and safety of MXene-embedded textiles are necessary due to their novelty. Until novel materials are proven reliable and cost-effective, established textile firms may be reluctant to invest in them.^282–285^ Next, another obstacle is the regulatory and environmental ones. MXenes’ possible toxicity, particularly if they break down or leak out during use or washing, raises safety concerns. Hazardous reagents are frequently used in chemical synthesis processes, and waste management can be challenging.^282–285^ Market introduction can be delayed by the time and expense required to navigate regulatory procedures (such as those for consumer electronics or wearable health devices). Overall, improving material stability through surface modifications or protective coatings, and focusing on developing scalable, cost-effective production techniques, are essential to easing the transition of MXene-based textiles from research and development to commercialization. After that, carry out thorough safety and durability testing, participate in early regulatory discussions to help establish standards, and invest in market outreach and education to increase demand and acceptance.^282–285^

**Figure 13:**
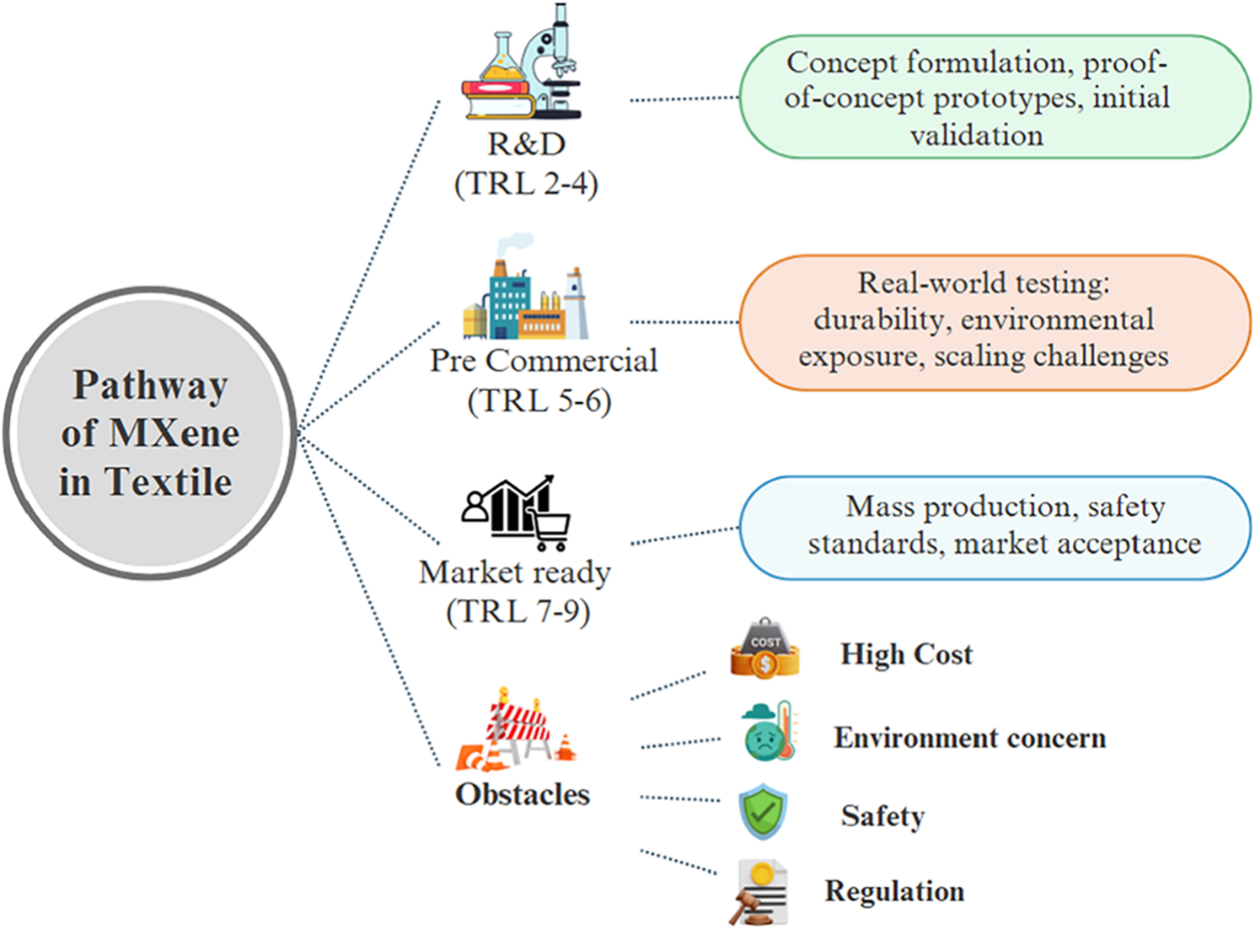
Pathway of MXene Technologies in Textiles (created with MS PowerPoint).

### 6.4 Compatibility with Textile Manufacturing

For MXenes to be commercially viable and used in the textile industry, it is essential to comprehend whether integrating them into current textile manufacturing processes is feasible. This section will examine the benefits and required modifications to existing production equipment, as well as other textile manufacturing processes where MXenes can be successfully integrated, including roll-to-roll processing, knitting, and printing, as shown in Figure 14. Roll-to-roll processing is one of the integration methods; it is a continuous manufacturing process in which textiles are unwound from a roll, treated, and then rewound. Applying MXene dispersions to fabric substrates, combined with polymer matrices, enables the integration of MXenes, improving the functionality and electrical conductivity of the fabric. High throughput is enabled by roll-to-roll processing, making it well-suited for scaling up the production of textiles infused with MXene. This process encourages the use of functional coatings, which enhance final product performance without sacrificing flexibility.^282–285^ Another is the knitting methods, which may incorporate MXenes into fibers with ease. For example, MXenes can be mixed with natural or synthetic fibers before knitting, preserving the stretchability and structural integrity of the functionalized yarns. This process strengthens the fabric’s overall strength and resilience while also improving electrical conductivity. Wearable technology and smart textiles may find new uses thanks to the versatility of knitted materials and the qualities of MXenes.^282–285^ The list also includes printing techniques.^282–285^ MXenes can be applied directly to textile surfaces using advanced printing methods such as screen printing or inkjet printing. Localized improvements, such as sensors or controllers embedded within the fabric, are enabled by these techniques, which allow the fabrication of patterned electrodes or functional zones.^282–285^ Printing processes provide manufacturing flexibility and design precision, enabling textile qualities to be customized for specific application needs. It is critical to have current equipment readiness. Many textile manufacturing machines on the market today are not explicitly designed to handle the unusual properties of MXenes, particularly regarding material compatibility and application procedures. Nonetheless, it may be possible to adapt devices such as coating machines, thermal calendars, and rotary screen printers with only very minor adjustments. For example, to control the viscosity and particle-size distribution of MXene dispersions, coating machines might need to be equipped with specialized nozzles or applicators.^282–285^ To maximize yarn tension and guarantee a steady incorporation of MXenes into the yarn structure, knitting machines might be necessary. Overall, the basic textile manufacturing machinery is adaptable to MXene integration; however, certain modifications might be required.^282–285^ New technologies and possible changes are also worrisome. Advanced mixing methods and dispersion and mixing techniques are needed to ensure that MXenes are uniformly distributed in polymer matrices or during yarn extrusion. Process control systems can help preserve the material’s functionality and quality throughout the manufacturing process by implementing real-time monitoring and feedback.^282–285^ The thermal stability of MXenes during and after printing or coating may require modifications to conventional drying or curing procedures, such as specialized dryers or curing ovens. In conclusion, there are great opportunities to create novel textile solutions by incorporating MXenes into traditional textile manufacturing processes. These materials’ ability to work with current methods, along with the required modifications, highlights their potential to revolutionize the textile sector by improving performance and functionality. By addressing both technical compatibility and manufacturing readiness, stakeholders can learn more about the best approaches to integrate MXene into textile production processes today successfully.^282–285^

**Figure 14:**
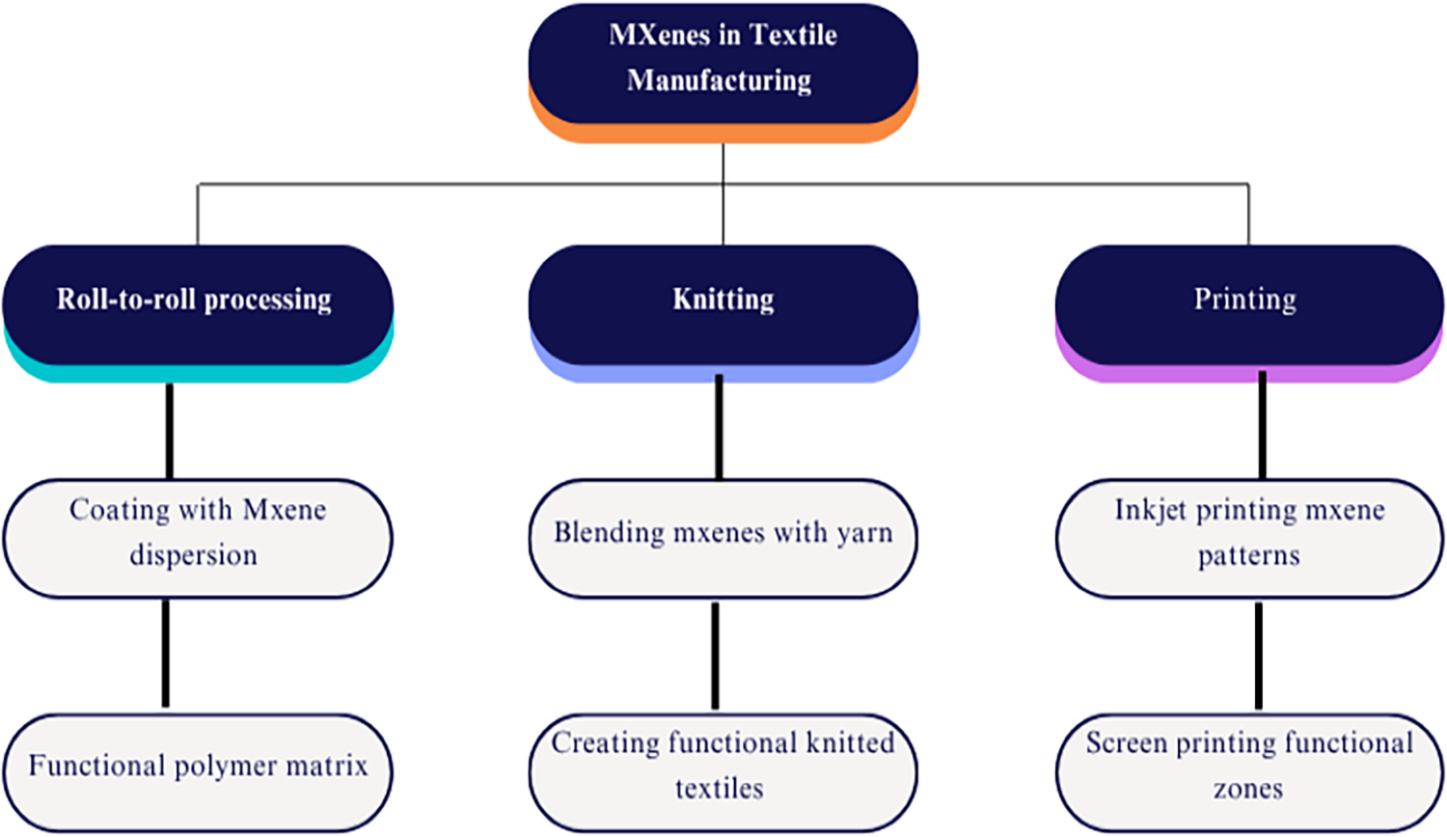
Pathway of MXene Technologies in Textiles (created with MS PowerPoint).

## 7 Future Opportunities and Research Directions

### 7.1 Multifunctional Textiles

Wearable technology is being transformed by the development of multifunctional fabrics that integrate sensing, power generation, and protective capabilities into a single fabric layer. Textiles that are not only flexible and breathable but also extremely conductive and responsive to environmental stimuli, thanks in significant part to developments in materials research, especially the integration of two-dimensional transition metal carbides and nitrides (MXenes). MXene-decorated fabrics, for instance, have been built with vertically aligned conductive networks that enable integrated Joule heating, electromagnetic interference (EMI) shielding, and strain sensing, all without compromising comfort or wearability. Researchers have precisely tuned the degree of conductive interconnectivity by varying the spray-drying cycles during the MXene coating process, producing fabrics with exceptional electrical conductivity (as low as 5 Ω sq^–1^ at a low MXene loading of 6 wt %), superior Joule heating performance (up to 150 °C at 6 V), excellent EMI shielding, and highly sensitive strain responses to human motion.^286^ MXene-based triboelectric nanogenerators (TENGs) capture biomechanical energy to power integrated sensors, thereby eliminating the need for external power.^287^ MXene-decorated polymeric textiles have shown dual-energy-driven heating (electrothermal and photothermal), a wide temperature range (40–174 °C electrothermal, 40–204 °C photothermal), fast thermal response (reaching over 100 °C within 25 s at 2.5 V), and additional functionalities such resistance to fire and bacteria, and high EMI shielding efficiency (42.1 dB in the X-band).^288^ Thus, these materials emphasize their adaptability for future health management and protection, as they are suitable for warmth retention, thermotherapy, deicing, heating water, EMI shielding, and antibacterial and fire protection.^288^ MXenes combined with other nanomaterials, such as graphene and conductive polymers, improve the multifunctionality of these textiles even more by allowing them to store electrical energy, harvest energy from the surroundings or body movements, and even self-heal or adapt to changing conditions.^289,290^ Degradable MXene-based textiles for sustainable wearable electronics, where MXene-doped polylactic acid textiles (DMPTs) are produced via electrospinning, resulting in materials with high sensitivity (5.37/kPa), fast response time (98 ms), and good mechanical stability (over 6000 cycles), have also lately attracted attention. These DMPTs effectively break down in mild alkaline solutions, therefore meeting the increasing need for wearable technologies with environmental impact.^290^ Mechanical stability is addressed by salt-assisted assembly techniques, resulting in ultrathin MXene coatings on Kevlar and polyether ether ketone (PEEK) with 86.5 Ω/sq sheet resistance following 168 h of extreme washing.^291^ MXenes combined with cellulose-based materials have also produced sustainable, multifunctional composites with photothermal, electrothermal, biocidal, and piezoelectric properties that are suitable for uses in wound dressings, desalination, and pressure sensors tracking human movement.^289^ Further development of innovative production techniques that enable the smooth integration of several functionalities into a single fabric layer, as well as the scalability, durability, and cost-effectiveness of these multifarious textiles, provides continuous challenges. Future research directions include investigating new hybrid systems combining MXenes with other functional materials, developing advanced coating and printing techniques for mass production, and integrating smart textiles with wireless communication and energy harvesting systems. Next-generation wearable technologies that are not only smart and functional but also comfortable, durable, and sustainable are being developed, ultimately from the intersection of materials science, nanotechnology, and textile engineering. For functional textiles, MXene aerogels and aerogel fibers provide definite benefits (large surface area, adjustable conductivity, lightweight, robustness), enabling energy storage, intelligent sensing, and EMI shielding. Fabrication methods (sol–gel-based aerogels; wet spun Mxene/polymer fibers; direct extrusion or coating of conventional fibers with MXene aerogel layers); modification techniques (surface functionalization with −OH/–O/–F and polymer grafting; cross-linking and network formation; composites with polymers, CNTs, graphene, or ceramics; drying controls like supercritical CO_2_, freeze-drying, or reinforced ambient drying).^292–294^

### 7.2 Sustainable MXene Textiles

The search for sustainable materials in wearable technology has yielded notable progress in developing biodegradable substrates for MXene-based textiles and in the green synthesis of MXenes. Although conventional MXene synthesis sometimes requires hazardous chemicals and energy-intensive techniques, current efforts have focused on developing environmentally friendly solutions that reduce environmental impact and waste generation. To generate MXenes with high purity and outstanding electrical properties, researchers have investigated, for instance, green chemical pathways and plant-based solvents. MXenes combined with biodegradable and renewable materials such as cellulose have produced new composite materials that retain the special qualities of MXenes while leveraging the sustainability and biocompatibility of natural polymers.^289^ To replace synthetic polymers such as PET, researchers are also exploring biomass-derived substrates, including cellulose nanofibers and chitosan coatings. MXene-coated hemp fabrics, for instance, show full compostability and similar conductivity (∼1000 S·m^–1^) to synthetic counterparts.^21,295^ By 40–60%, circular design strategies, including solvent-free MXene dispersion processes and roll-to-roll manufacturing, also help lower water and energy use compared to traditional approaches.^295^ From wound dressings and pressure sensors to desalination and antimicrobial protection, these composites exhibit extraordinary properties, including photothermal and electrothermal conversion, biocidal activity, and piezoelectric effects, making them well-suited for a broad spectrum of applications. Demonstrating the promise for multifarious, sustainable textiles, the creative approach of in situ creation of zeolitic imidazolate framework-8 (ZIF-8) on the surface of cellulose nanofibers significantly increases the biocidal activity and EMI shielding performance of these materials.^289^ By electrospinning, which combines the biocompatibility and degradability of polylactic acid with the conductivity and sensing capabilities of MXenes, another exciting area is the creation of degradable MXene-doped polylactic acid textiles (DMPTs).^290^ These DMPTs may be effectively reduced in mild alkaline solutions, thereby addressing the growing need for environmentally friendly wearable electronics. They exhibit excellent sensitivity (5.37 kPa), a fast response time (98 ms), and strong mechanical stability (over 6000 cycles). MXene nanosheets also enhance the hydrophilicity and degradation efficiency of polylactic acid nanofibrous films, thereby improving their sustainability.^290^ Combining MXenes with renewable resources such as cellulose and polylactic acid not only reduces the environmental impact of wearable textiles but also creates new opportunities for developing innovative, biodegradable devices for environmental sensing, personal protection, and health monitoring.^289,290^ Future work should focus on optimizing green synthesis methods for MXenes, enhancing the scalability and cost-effectiveness of biodegradable MXene-based textiles, and investigating novel applications in medicine, agriculture, and environmental monitoring. The next generation of wearable devices is intended to be not only innovative and functional but also environmentally responsible and socially acceptable by combining sustainable materials with advanced features.

### 7.3 IoT and AI Integration

Wearable textiles combined with artificial intelligence (AI) and the Internet of Things (IoT) are transforming the world of bright clothing by enabling real-time data collection, analysis, and decision-making across a broad spectrum of applications. The high conductivity, flexibility, and multifunctionality of MXene-based textiles make them ideal platforms for embedding sensors, energy harvesters, and communication modules into apparel.^286,288^ These bright clothes may wirelessly send a range of physiological and environmental data, such as body temperature, heart rate, movements, and electromagnetic field exposure, to cloud-based artificial intelligence systems for real-time monitoring and analysis.^286,288^ MXene-decorated fabrics with integrated strain sensing, for instance, may identify minute motions and gestures, therefore offering helpful feedback for sports performance, rehabilitation, and fall detection. MXene-based sensors, coupled with IoT connectivity, enable continuous, remote monitoring of health parameters, facilitating early diagnosis of medical conditions and tailored treatments.^286^ Combining IoT and artificial intelligence with MXene fabrics yields flexible, innovative systems for environmental sensing and real-time health monitoring. 8% accuracy in identifying hand tremors and rigidity, and subsequently, Bluetooth Low Energy (BLE) data is delivered to mobile apps for clinical analysis.^296^ MXene-based RFID tags spun into textiles allow real-time inventory tracking through automated stock updates, therefore reducing supply chain waste by 20%. Using biomechanical data enables machine learning algorithms, including convolutional neural networks (CNNs), to improve the accuracy of pressure prediction (<5% error) in MXene piezoresistive sporting gear.^297^ Using reinforcement learning, MXene PEDOT and PSS fabric-powered artificial intelligence-powered thermal management systems dynamically adjust protection based on environmental temperature, therefore maintaining skin temperature within ±0.5 °C even in changing outside conditions.^298^ By enabling cooperative data processing across distributed MXene sensor networks, federated artificial intelligence systems help to improve the accuracy of air quality monitoring in smart cities by 35%.^299^ MXene hydrovoltaic generators, embedded in textiles, gather ambient humidity to drive IoT nodes, enabling 13 min of wireless operation after 30 min charge.^300^

### 7.4 Predictive Modeling and Optimization

Predictive modeling and machine learning (ML) methods substantially accelerate the design and optimization of MXene-based textile constructions for specific applications. Although conventional trial-and-error methods for material development are time-consuming and resource-intensive, ML algorithms can analyze large data sets of material properties, processing parameters, and performance criteria to identify optimal pairings for the desired functionality. Based on variables like MXene loading, coating technique, and substrate type, ML models may forecast, for instance, the mechanical strength, electrical conductivity, and sensing capability of MXene-textile composites.^290^ To achieve the optimal balance among conductivity, flexibility, and durability, these models can also optimize the fabrication process via electrospinning settings or spray-drying cycles.^286,290^ MXene-textile composites are being designed revolutionarily by machine learning (ML) and quantum computing technologies. Targeted surface functionalization enabled by density functional theory (DFT)-guided models predict interfacial bonding strengths between MXene flakes and polyamide substrates, hence improving wash durability.^301^ Degradable MXene-doped polylactic acid textiles (DMPTs), where the combination of polydimethylsiloxane templating and MXene flake impregmentation methods was optimized to achieve high sensitivity (5.37/kPa), fast response time (98 ms), and good mechanical stability (over 6000 cycles), have shown the use of ML-driven design tools. For MXene-based textiles, predictive modeling can also guide the selection of biodegradable substrates and green synthesis pathways, thereby ensuring that the materials are not only highly performing but also sustainable and environmentally benign.^289,290^ Moreover, ML techniques may analyze wearable sensor data to identify trends, predict failures, and optimize maintenance plans for innovative clothing.^290^ Next-generation wearable gadgets adapted to particular user needs, applications, and environmental circumstances are expected to be developed using the integration of ML with materials science and textile engineering. The development of improved ML models for multiobjective optimization, the integration of real-time data from wearable sensors, and the investigation of new materials and processing techniques should take center stage in future work. Taken together, predictive modeling, ML, and cutting-edge textile materials should transform smart garment design, manufacturing, and performance for environmental sensing, personal protection, and health monitoring.

### 7.5 Novel Hybrid Systems

New opportunities for greater usefulness in wearable textiles are being created by the development of unique hybrid systems that combine MXenes with other advanced materials, such as metal–organic frameworks (MOFs), proteins, and ceramics. Integrated with MOFs, MXenes, with their special mix of metallic conductivity, hydrophilicity, and programmable surface chemistry, can be created with enhanced sensing, catalytic, and filtering characteristics. MXene hybrid systems enable multifunctionality in wearable textiles by synergizing with metal–organic frameworks (MOFs), proteins, and ceramics. Conductive MXene frameworks supporting MOF nanostructures provide better electrochemical stability (overpotential of 52 mV at 10 mA/cm^2^ for hydrogen evolution) in MXene-MOF composites, including Ti_3_C_2_T*_x_*/ZIF-8.^290,302^ Protein- MXene composites, such as bovine serum albumin (BSA)-Ti_3_C_2_T*_x_*, self-assemble into topological networks in biomedical textiles, therefore providing variable interlayer spacing (0.92–1.2 nm) for drug delivery or strain sensing.^303,304^ By leveraging MXene’s mechanical resilience, ceramic hybrids such as Al_2_O_3_/Ti_3_C_2_T*_x_* improve fracture toughness by 40% in aerospace textiles.^305^ MXene silver cellulose nanofiber composites achieve 43× higher conductivity than pure MXene membranes for energy storage, thereby enabling foldable supercapacitors with 396 F/g capacitance.^26^ Furthermore, addressing environmental issues through hybridization: MXene-polycaprolactone (PCL) nanofibers functionalized with aspirin reduce reactive oxygen species (ROS) by 60%, thereby accelerating wound healing in smart bandages.^306^ Furthermore, enabling UV-resistant e-textiles with 95% EMI shielding efficiency are MXene-quantum dot hybrids buried in polydimethylsiloxane (PDMS). These hybrids show how material synergies can go beyond the constraints of individual components to produce adaptable systems for aircraft, energy, and healthcare.

### 7.6 Cross-Disciplinary Research

Wearable e-textiles based on MXenes and related materials advance using the synergy of cross-disciplinary research, combining knowledge from materials science, textile engineering, biomedical engineering, and data science. Cooperation across many disciplines helps to create creative materials with customized features, cutting-edge production methods, and integrated sensing, power, and communication capabilities.^286,288^ For instance, while textile engineers provide scalable production techniques and guarantee the comfort, durability, and wearability of bright clothing, materials scientists help to design and synthesize MXenes and hybrid composites. Data scientists provide algorithms for real-time data analysis, predictive modeling, and personalized health monitoring; biomedical engineers concentrate on the biocompatibility, safety, and clinical value of wearable devices. MXene-based textiles for health monitoring, in which sensors, energy harvesters, and communication modules are subtly integrated into apparel for continuous, noninvasive monitoring of physiological and environmental parameters, epitomize the integration of these disciplines. Additionally, driving the creation of sustainable, biodegradable fabrics for medicinal and ecological uses, as well as the integration of IoT and artificial intelligence technologies for bright, adaptive clothing, is a cross-disciplinary collaboration.^289,290^ MXene-based textiles are inspiring innovation by converging material science, textile engineering, biomedicine, and data science. Wearable MXene sensors linked with edge-computing processors, for example, categorize full-body motions with 100% accuracy, thereby combining materials design (wrinkle-topography MXene nanolayers) with machine learning (ANN models).^307,308^ MXene-hydrogel composites combine the biocompatibility of HA-DA hydrogels with the photothermal activity of MXene to reduce the time required for diabetic wound healing by 50% through regulation of the HIF-1α pathway. Predictive toxicology is enabled by data science; federated learning models evaluating MXene cytotoxicity across 15 institutions found that F-terminated Ti_3_C_2_T*_x_* was the least inflammatory (cell death <5%).^309,310^ Working with chemists, textile engineers create plasma-treated MXene-cotton fabrics whose oxygen functional groups improve MXene adherence by 300%, hence enabling wash-resistant ECG electrodes.^26^ Concurrently, sustainability projects employ artificial intelligence to maximize MXene recovery from textile waste using solvent selection algorithms, thereby achieving 90% purity. Such joint efforts highlight the need for comprehensive solutions for complex problems in scalability, biocompatibility, and integration of the circular economy. Sericin modification provided superior protection against oxidative degradation. One major obstacle to dependable device performance is MXene vulnerability to atmospheric and aqueous oxidation. A straightforward biocompatible method for passivating reactive MXene sites and slowing oxidative development without compromising electrochemical activity is sericin-based surface modification. Sericin offers advantages in processability, environmental friendliness, and compatibility with aqueous processing compared to other stabilizing methods (such as iron oxide coating and polymer grafting). According to our findings, sericin-modified MXene retains charge storage properties and structural integrity under oxidative stress, indicating longer device lifetimes in real-world applications. Further research should clarify the molecular function of sericin at the MXene surface (e.g., shielding reactive terminators, producing protective interfaces) as well as evaluate long-term stability across electrolytes and temperatures^311,312^

## 8 Conclusion

MXene-based multifunctional and biomedical smart textiles are opening new possibilities in wearable technology by combining outstanding electrical conductivity, flexibility, and tunable surface chemistry with the comfort and versatility of traditional fabrics. These materials have shown great promise in applications such as highly sensitive health monitoring sensors, self-powered energy systems, EMI shielding, thermal regulation, antimicrobial protection, and advanced communication features. With practical integration methods such as dip-coating, printing, and layer-by-layer assembly, MXenes can be used to create lightweight, durable, and responsive e-textiles that still feel comfortable to wear while delivering advanced performance.

To move from lab research to real-world products, essential challenges must be addressed, including improving oxidation resistance, ensuring durability after repeated use and washing, confirming biocompatibility, and developing cost-effective large-scale manufacturing processes. Solutions will involve protective coatings, hybrid material designs, environmentally friendly synthesis methods, and thorough safety testing. Progress will also depend on close collaboration among materials scientists, textile engineers, biomedical experts, and data specialists to integrate MXenes into intelligent systems such as IoT and AI. If these steps are taken, MXene-integrated textiles could become a key part of the next generation of smart, sustainable, and user-friendly wearables for healthcare, defense, and consumer markets.
